# Fine-tuning of chromatin composition and Polycomb recruitment by two Mi2 homologues during *C. elegans* early embryonic development

**DOI:** 10.1186/s13072-016-0091-3

**Published:** 2016-09-15

**Authors:** Stéphanie Käser-Pébernard, Catherine Pfefferli, Caroline Aschinger, Chantal Wicky

**Affiliations:** 1 Biology Department, Zoology Institute, University of Fribourg, Ch. du musée 10, 1700 Fribourg, Switzerland; 2 Biology Department, Biochemistry Institute, University of Fribourg, Ch. du musée 10, 1700 Fribourg, Switzerland

## Abstract

**Background:**

The nucleosome remodeling and deacetylase complex promotes cell fate decisions throughout embryonic development. Its core enzymatic subunit, the SNF2-like ATPase and Helicase Mi2, is well conserved throughout the eukaryotic kingdom and can be found in multiple and highly homologous copies in all vertebrates and some invertebrates. However, the reasons for such duplications and their implications for embryonic development are unknown.

**Results:**

Here we studied the two *C. elegans* Mi2 homologues, LET-418 and CHD-3, which displayed redundant activities during early embryonic development. At the transcriptional level, these two Mi2 homologues redundantly repressed the expression of a large gene population. We found that LET-418 physically accumulated at TSS-proximal regions on transcriptionally active genomic targets involved in growth and development. Moreover, LET-418 acted redundantly with CHD-3 to block H3K4me3 deposition at these genes. Our study also revealed that LET-418 was partially responsible for recruiting Polycomb to chromatin and for promoting H3K27me3 deposition. Surprisingly, CHD-3 displayed opposite activities on Polycomb, as it was capable of moderating its LET-418-dependent recruitment and restricted the amount of H3K27me3 on the studied target genes.

**Conclusion:**

Although closely homologous, LET-418 and CHD-3 showed both redundant and opposite functions in modulating the chromatin environment at developmental target genes. We identified the interplay between LET-418 and CHD-3 to finely tune the levels of histone marks at developmental target genes. More than just repressors, Mi2-containing complexes appear as subtle modulators of gene expression throughout development. The study of such molecular variations in vertebrate Mi2 counterparts might provide crucial insights to our understanding of the epigenetic control of early development.

**Electronic supplementary material:**

The online version of this article (doi:10.1186/s13072-016-0091-3) contains supplementary material, which is available to authorized users.

## Background

Throughout embryonic development, gene expression must be dynamically regulated in a time- and cell-specific manner to promote proper lineage commitment, tissue formation, and growth. Epigenetic factors transiently modify the chromatin structure or composition to modulate gene expression in a rapid and reversible manner. The nucleosome remodeling and deacetylase complex (NuRD) appears as a central player during early embryogenesis. Its transcriptional repression activities are required to maintain pluripotency and to respond properly to developmental cues. NuRD is essential for early embryonic development [1, 2], for pluripotency maintenance, and for proper lineage commitment [3–5]. In mammals, NuRD is a high molecular weight complex composed of a Mi2 core SNF2-like ATPase/helicase subunit, HDAC1/2 histone deacetylases, methyl-cytosine binding domain (MBD) proteins MBD2/3, as well as structural or scaffolding proteins including the metastasis tumor antigen (MTA) 1/2/3, the GATAD2A or 2B (p66α/β) factors, and retinoblastoma binding proteins RBBP-4 or 7 (reviewed in [6–8]). The H3K4me2/3 histone demethylase LSD1 is also considered as a *bona*-*fide* NuRD member [9]. The combinatorial assembly of these subunits is thought to drive distinct biochemical or tissue specificities to NuRD complexes. The three vertebrate MTA family members evolved from successive genome duplications (reviewed in [7]) and are oppositely involved in mediating the response to the estrogen receptor (ER) in mammary tissue growth and during breast cancer invasion [8], suggesting that they achieve tissue-specific functions. The MBD2/3 DNA-binding proteins are mutually exclusive in NuRD complexes and associate with both common and distinct partners in mammalian cells [10]. Vertebrates count two-to-three Mi2 family members, namely CHD3, CHD4 and/or CHD5. Mammalian CHD5, a putative tumor suppressor, is mostly expressed in the nervous system and the intestine, whereas CHD3 and CHD4 are almost ubiquitous [11, 12]. In *zebrafish*, a regeneration-specific NuRD complex including Chd4a, but not Chd4b or Chd3, assembles specifically after fin amputation to promote osteoblast differentiation and fin regeneration [13]. In the model organisms *A. thaliana*, *C. elegans* and *D. melanogaster*, two-to-three Mi2 homologues were identified. *A. thaliana* expresses three Mi2 homologues, namely PICKLE (*Pkl*) and PICKLE-related proteins 1 and 2 (*Pkr1* and *Pkr2*) [14], and *pkl* and *pkr2* show redundant functions in suppressing embryonic lethality and establishing cell identity [15]. *Drosophila* dMi2 and dChd3 are structurally different, since dChd3 misses N- and C-terminal regions as well as one PHD finger and is incapable of replacing dMi2 functions [16]. *C. elegans* LET-418 and CHD-3 proteins are 70 % identical and 86 % similar. Although *chd*-*3* depleted worms show no obvious phenotype, and *let*-*148* depletion leads to L1 arrest, double *let*-*418; chd*-*3* depletion leads to embryonic lethality, suggesting a redundant function of *let*-*418* and *chd*-*3* in early development [17]. In addition, *let*-*418* and *chd*-*3* act redundantly to promote proper differentiation of the vulval cell precursors P5.p and P7.p, implying that common functions of these proteins are required at later developmental stages and adulthood [17].

To understand the reasons for maintaining multiple Mi2 copies in eukaryotic organisms, we decided to analyze the functions of both LET-418 and CHD-3 in *C. elegans*. We identified a gene subset redundantly and directly repressed by the two Mi2 homologues in early embryos. Surprisingly, we found that LET-418 and CHD-3 differentially regulated histone modifications at the promoter of these genes. While both proteins restrict H3K4 methylation, LET-418 is capable of recruiting the H3K27 methyltransferase Polycomb to target genes promoters and CHD-3 limits this recruitment. We propose a model for the fine-tuning of chromatin composition and structure by a well-regulated balance between two Mi2 homologues, which potentially could constitute a conserved mechanism in all eukaryotes retaining multiple Mi2 copies.

## Results

### Mi2 duplications are ubiquitous in vertebrates, less frequent in invertebrates

**Fig. 1:**
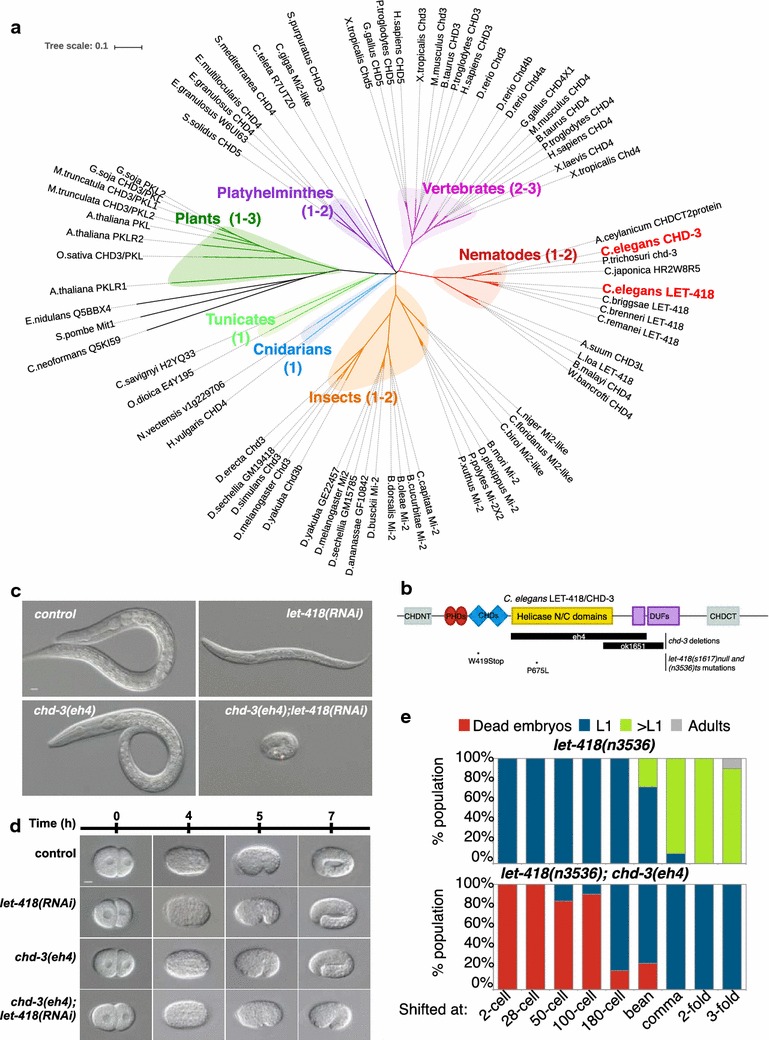
Mi2 *C. elegans* LET-418 and CHD-3 homologues display redundant functions in early embryonic development. **a** Phylogenetic tree of Mi2 homologues in different eukaryotic phyla. **b** Schematic structure of the *C. elegans* Mi2 homologues LET-418 and CHD-3. Domain abbreviations: CHDN/CT, CHD-N- or C-terminal domain; PHD, plant homology domains; CHDs, chromodomain, helicase and DNA binding; helicase domains: N- and C-terminal helicase domains, associated with DEXDc family; DUF: domain of unknown function. Mutations used in this study are indicated as deletions (*black rectangles*) or point mutations (*asterisk*). **c**, **d** Nomarski images of wild-type or *chd*-*3(eh4)* worms treated with *control* or *let*-*418(RNAi)*, 24 h post-egg laying at 25 °C (**c**) or at the indicated time points (**d**). *Scale bar* 10 μm. **e** Redundant functions of LET-418/CHD-3 are required before the 100-cell stage. *let*-*418(n3536)* (*upper graph*) or *let*-*418(n3536); chd*-*3(eh4)* (*lower graph*) embryos were shifted at 25 °C to inactivate LET-418 activity at the indicated embryonic stages, and analyzed for their phenotypes. Between 19 and 33 embryos were tested per condition

Besides *C. elegans* and *D. melanogaster*, many eukaryotic organisms express more than one Mi2 family member, most particularly in vertebrate species. Phylogenetic analysis performed on a selection of eukaryotic Mi2 homologues implies that they developed individually from a common ancestor and that duplications occurred independently within subkingdoms (Fig. 1a). In plants, three Mi2 homologues are known for *Arabidopsis*, and we identified two putative CHD3 homologues in soybean (*G. soja*) and barrel clover (*M. truncatula*), but not in common rice (*O. sativa*) (Fig. 1a). Among invertebrates, we could only identify Mi2 duplication in the nematode *C. elegans*, a putative duplication in the tapeworm *E. granulosus*, as well as in multiple species of the *Drosophila* genus, but not in any other insect species (Fig. 1a). In species belonging to other invertebrate phyla, including *Cnidaria*, *Tunicata*, *Mollusca*, *Annelida*, or *Echinodermata*, we could only identify one Mi2 copy, although the number of fully sequenced species was limited (Fig. 1a). The observation that most invertebrate species, from a variety of phyla, display only one Mi2 copy, suggests that the duplications that occurred in *C. elegans* and in the *Drosophila* genus were isolated events. In contrast, most vertebrates evolved two-to-three Mi2 copies (CHD3, CHD4 and/or CHD5), suggesting that Mi2 duplications occurred early in the vertebrate kingdom; noticeably, CHD-5 seems to have derived from a common ancestor to CHD-3 (Fig. 1a). Therefore, each subkingdom in the eukaryotic reign probably evolved and developed its own Mi2 system.

### The two *C. elegans* Mi2 homologues function redundantly in early embryonic development

The frequent Mi2 duplications in more complex organisms, such as vertebrates, evoke the requirement for a more advanced system of developmental control. To understand the significance and functions of Mi2 duplications in controlling eukaryotic development, we took advantage of the duplication existing in the model organism *C. elegans*. Its two Mi2 proteins, CHD-3 and LET-418, share a high degree of sequence homology, with 71 % identity and 80 % similarity (BLASTP). Mi2 proteins are characterized by specific functional domains, including tandem N-terminal chromodomains and DNA binding (CHD) and plant homeo-domains (PHDs), and central SNF2-like ATPase and DEAD-like helicase domains (Fig. 1b, for reviews see [6, 18]). LET-418 and CHD-3 show high domain conservation, particularly in the ATPase and Helicase domain (Additional file 1: Figure S1A). To identify functional differences between the two homologues, we studied the effects of combined *chd*-*3 null* (*eh4 or ok1651*) mutations with *let*-*418 (RNAi)* or with a temperature-sensitive (ts) *let*-*418(n3536)* allele, on *C. elegans* development (Fig. 1b). To avoid the effect of hidden mutations that might have escaped back-crossing, we analyzed the phenotype of worms with different combinations of *chd*-*3* alleles with *let*-*418* mutations or *let*-*418(RNAi)* (Fig. 1c–e, Additional file 1: Figure S1C, D). As expected, loss of maternal *let*-*418* expression led in all cases to a fully penetrant L1 arrest of the progeny, whereas *chd*-*3 null* worms grew as wild-type ([17, 19, 20], Fig. 1c–e; Additional file 1: Figure S1B, C). Double *let*-*418; chd*-*3* embryos, generated either by exposing *chd*-*3 null* L4 mothers to *let*-*418(RNAi)* or by shifting *chd*-*3(eh4); let*-*418(ts)* mothers to restrictive temperature, showed a visible growth delay from the bean stage on, and a developmental arrest at the twofold stage (Fig. 1d, e; Additional file 1: Figure S1C-D). The embryonic lethality of double *let*-*418; chd*-*3* mutants suggests a redundant function of *chd*-*3* in absence of *let*-*418* during early development.

### LET-418 and CHD-3 redundantly repress a specific gene subset in early embryogenesis

**Fig. 2:**
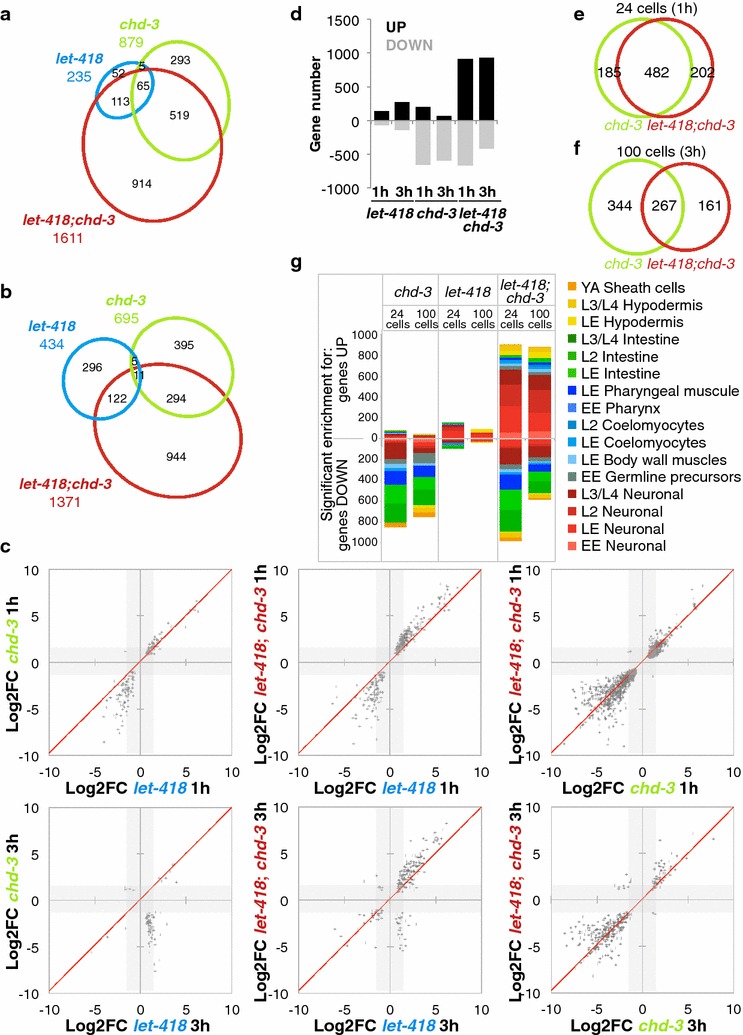
Transcriptome analysis of *let*-*418; chd*-*3* double mutants identifies a gene population redundantly regulated by the two Mi2 homologues. **a**, **b** Venn Euler diagrams showing the total number of deregulated genes in each mutant genotype (wild-type on *let*-*418(RNAi)*; *chd*-*3(eh4)* on *control(RNAi)*; *chd*-*3(eh4)* on *let*-*418(RNAi)*) at the 24-cell (**a**) and 100-cell (**b**) stages, relative to *control(RNAi)*-treated wild-type embryos. List of genes deregulated more than 1.5 fold (log2 fold change) with a *p* value <0.1 were used. Common genes *p* value <0.01. **c** *Dot plots* of common deregulated genes between the indicated genotypes at 1 h (24-cell) or 3 h (100-cell) stages. Genes deregulated below the threshold Log2FC of 1.5 are displayed in *gray shaded areas*. **d** Proportion of up- and down-regulated genes in each RNA-Seq sample. **e**, **f** Venn Euler diagrams of commonly down-regulated genes in *chd*-*3(eh4)* mutants treated with *control* or *let*-*418(RNAi)* at the 24-cell (**e**) or 100-cell (**f**) stage. *p* values of common genes <0.01. **g** Cumulative enrichment of RNA-Seq up- or down-regulated genes in tissue- and stage-specific genes of the Spencer lists. Only enrichments with significant *p* value (<0.01) were scored. All enrichments were combined in one column per sample, but a same gene can be present in multiple lists. *EE* early embryo, *LE* late embryo, *YA* Young adults

Blastomeres are known to remain developmentally plastic until the onset of gastrulation (24-cell stage) and to undergo lineage establishment until the 100-cell stage [21]. Importantly, we determined that the redundant functions of LET-418 and CHD-3 were required within this period of developmental plasticity, since inactivation of LET-418 after the 100-cell stage did not lead to embryonic lethality (Fig. 1e; Additional file 1: Figure S1D). To identify the target genes involved in the *let*-*418; chd*-*3* embryonic lethality, we therefore decided to analyze the transcriptome of Mi2 mutants during this plasticity period. Single *let*-*418(RNAi)* and *chd*-*3(eh4)* or double *chd*-*3(eh4); let*-*418(RNAi)* embryos were manually sorted and collected at the 24- and 100-cell stages, and compared to *wild*-*type; control GFP(RNAi)*-treated embryos in a transcriptome RNA sequencing (RNA-Seq) analysis (Fig. 2). Genes significantly deregulated (*p* value <0.1, −1.5 > Log2FC > 1.5) in single- and double-mutant embryos were investigated (see Additional file 3: Additional Spreadsheet for all deregulated genes lists), and the RNA-Seq experiment was validated by quantitative RT-PCR on a gene subset (Fig. 2; Additional file 1: Figure S2B-D). No chromosome bias was observed in this study (Additional File 1: Figure S2A). Double *let*-*418; chd*-*3* embryos displayed more deregulated genes than single mutants at both 24- and 100-cell stages, confirming the redundant functions of LET-418 and CHD-3 on gene expression (Fig. 2a–c). Most particularly, many genes induced in absence of a functional *let*-*418* saw their induction increased in double *let*-*418; chd*-*3* mutants (Fig. 2c). In total, 94 and 86 % genes common to *let*-*418* and *let*-*418; chd*-*3* lists at the 24- and 100-cell stages, respectively, were up-regulated to higher levels in double mutants, whereas common genes of the *chd*-*3* and *let*-*418; chd*-*3* lists did not show more deregulation in double versus single mutants (Fig. 2c). Cross-comparisons with tissue-specific gene datasets showed that genes up-regulated in *let*-*418* embryos associated with a few neuron-specific genes, while genes up-regulated in double *let*-*418; chd*-*3* mutants associated largely with neuron-specific datasets [22] (Fig. 2g), indicating that CHD-3 redundantly repressed neuronal genes in absence of *let*-*418*. A small number of pharyngeal, body wall muscle, and intestinal genes at both the 24- and 100-cell stages, and hypodermal genes at the 100-cell stage, also appeared to be redundantly regulated by LET-418 and CHD-3 (Fig. 2g). A large fraction of the deregulated neuronal genes were specific to later embryonic or larval stages (Fig. 2g), suggesting that neuronal cell markers were expressed prematurely in Mi2 double mutants.

Surprisingly, both *chd*-*3* and *let*-*418; chd*-*3* embryos displayed a significant amount of down-regulated genes, which was not expected considering the transcriptional repressor role of NuRD (Fig. 2c, d). This down-regulated gene subset was for a significant fraction common to the two mutants (most particularly at the 24-cell stage, Fig. 2e, f) associated principally with intestinal, body wall muscle, and pharyngeal genes (Fig. 2g) and did not increase in number in the double mutants, implying that it was only controlled by *chd*-*3* (Fig. 2g). Taking into account that *chd*-*3 null* mutants do not show any visible phenotype, we hypothesized that the down-regulation of this gene population nevertheless did not impact embryonic development. However, the occurrence of a population of down-regulated genes in the absence of *chd*-*3* activity already points out a specialized function of CHD-3 compared to LET-418.

### LET-418 enriches at a TSS-proximal location on target genes promoter

To characterize the molecular mechanisms of Mi2 control on gene expression, we aimed at identifying direct target genes. A ChIP-Sequencing (ChIP-Seq) analysis of LET-418 genome-wide binding sites was performed in duplicate embryonic extracts, using a fully functional LET-418::3xFLAG transgene expressed in a *let*-*418(ts)* background (Additional file 1: Table 1). As we aimed at comparing these assays to the RNA-Seq data we already generated, but single embryonic stage ChIP was not technically feasible, early-stage embryos were enriched in these populations by two sequential synchronizations. Young adults were harvested for embryo extraction, and a fraction of around 75 % pre-bean stage (<350 cells, end of gastrulation) embryos was confirmed at cross-linking.

**Fig. 3:**
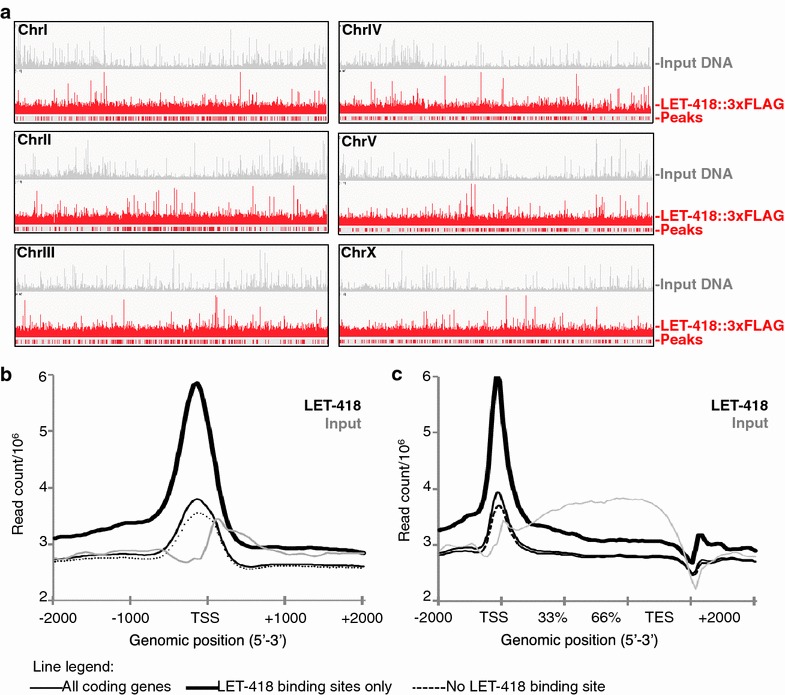
LET-418 associates with chromatin genome-wide and is enriched proximally to the TSS. **a** Genome-wide ChIP-Seq binding profile of embryonic LET-418::3xFLAG (*red lines*), compared with non-enriched input DNA (*gray*). *Red bars* below: LET-418 most enriched sites (MACS2 peaks). *Scale* 0–163 (composite value). **b**, **c** Ngsplot of LET-418 genome-wide enrichment centered on the transcription start site (TSS) (**b**), or on the gene body between TSS and TES (**c**). Input DNA profile is represented in *gray* for comparison. Ngsplot profiles were calculated for the entire coding-gene population, or only for the list of LET-418 enriched (peaks) or non-enriched genes. *Y* axis: read count per million mapped reads

Anti-FLAG ChIP-Seq demonstrated that embryonic LET-418 was spread genome-wide at a basal level (Fig. 3a). 3904 regions of most significant LET-418 enrichment, normalized to the input DNA, were identified (“peaks”) and were visibly condensed at the center of all chromosomes (Fig. 3a), consistent with an association to “open” transcriptional zones at the center of chromosomes [23]. Peaks were more abundant on chromosome V, reflecting the highest amount of ORFs on this autosome (Additional file 1: Figure S3A and www.Ensembl.org) and were broad, with an average size of approximately 2000 bp (Additional file 1: Figure S3B). Total base-pair coverage of significant peaks did not exceed 9 % of the total chromosome size (Additional file 1: Figure S3C). Using the ngsplot tool [24] to position LET-418 genome-wide reads relative to the gene structure, we observed that our biological duplicate ChIP-Seq samples showed similar enrichment patterns, and hence only one of the duplicates was used in further studies (Additional file 1: Figure S3D, E). We observed that LET-418 associated preferentially with a transcriptional start site (TSS)-proximal location (Additional file 1: Figure S3D–E), with peak binding intensity at −160 bp from TSS, and higher enrichment level for LET-418 most significant peaks reads (Fig. 3b, c). Altogether, our data show that, although generally widespread at basal levels on chromatin, LET-418 preferentially accumulates at a TSS-proximal site on a subset of target genes.

### LET-418 associates with histone marks of transcriptionally active gene chromatin

**Fig. 4:**
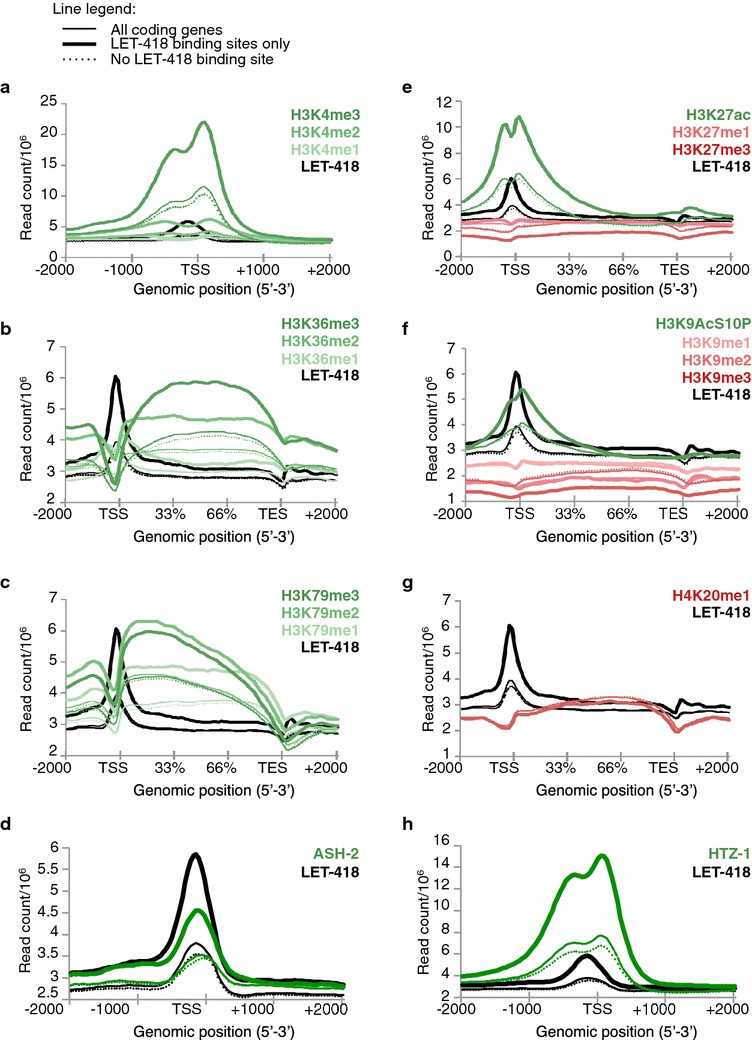
LET-418 binding sites are enriched in activating chromatin marks genome-wide. **a**–**c**, **e**–**g** Ngsplot genome-wide binding profiles of embryonic LET-418 compared with the indicated histone marks, for the entire coding-gene population, or for the list of LET-418-enriched (peaks) or non-enriched (no peak) genes. Plots were calculated relative to the TSS or gene body position, depending on the histone mark. Activating histone marks are represented with *green lines*, whereas repressive marks are represented with *red lines*. **d**, **h** Ngsplot of LET-418 and embryonic ASH-2, a COMPASS component (**d**), or the transcriptional activator histone variant HTZ-1 (**h**), relative to the TSS position. *Y* axis: read count per million mapped reads

The LET-418 pre-TSS accumulation could be important for modulating the chromatin environment at the TSS and thus to influence transcription. To characterize the chromatin status at LET-418- enriched sites, the LET-418 genomic position profile was compared with profiles of embryonic histone marks, which we processed from the embryonic ChIP-Seq data available in the ModENCODE database [25]. LET-418 most significant target genes were enriched for histone marks associated with active transcription, such as H3K4me2/3, H3K36me2/3, H3K79me2/3, H3K9AcS10P and H3K27ac (Fig. 4a–c, e and reviewed in [26–31]), as well as HTZ-1, a histone variant associated with active gene TSS [32] (Fig. 4h). Strikingly, the LET-418 pre-TSS peak coincided with a “trough,” observed for each histone mark or variant profile (Fig. 4), which could correspond to the “nucleosome depleted region” (NDR) characteristic of transcriptionally active genes [33, 34]. The LET-418 most significant target genes were also enriched in ASH-2, a COMPASS H3K4 methyltransferase member [35], and in this case the LET-418 and ASH-2 peaks aligned together at the NDR putative region (Fig. 4d), suggesting that multiple chromatin remodelers could locate to the pre-TSS NDR. The H3K27me3 and H3K9me3 marks, associated with gene silencing and heterochromatin formation [36], were depleted on LET-418 most significant peaks (Fig. 4e, f). Conversely, genes marked by H3K27me3 were less occupied by LET-418 than unmarked genes (Additional file 1: Figure S4). Finally H4K20me1, a histone mark associated with negatively regulated genes [37], did not vary on LET-418-enriched genes (Fig. 4g). Our observations show that LET-418 accumulates at a TSS-proximal location of transcriptionally active genes and is depleted on inactive and heterochromatic genes. The LET-418 protein contains tandem chromodomains, which are known binders of methylated histones [38] and could be responsible for LET-418 accumulation at these loci, although the affinity of LET-418 chromodomains for activating histone marks remains to be demonstrated. Most interestingly, the presence of repressive Mi2 complexes at TSS-proximal sites could serve to modulate the chromatin context and the transcriptional activity of their target genes. Indeed, our findings correlate with a number of chromatin binding studies in mammalian cells that have shown transcriptional repressor complexes to be associated with active gene loci to act as fine-tuners of gene expression during development (reviewed in [39]). Mi2 proteins, in particular in organisms where they are duplicated, could be used at TSS-proximal genes to modulate chromatin composition and accessibility to transcriptional machineries. This would be of particular importance in the early phases of embryonic development, in which cellular plasticity must by maintained by tuning down developmental gene expression. Indeed, gene ontology (GO) term analysis of genes bound at their promoter by LET-418 revealed an enrichment for the processes of growth regulation, post-embryonic development, and reproduction (Additional file 1: Table 2), consistent with a role of Mi2 complexes in promoting early embryonic developmental processes and maintaining a degree of plasticity by controlling the expression of key developmental and growth genes.

### LET-418 and CHD-3 differentially regulate histone mark levels on target gene chromatin

**Fig. 5:**
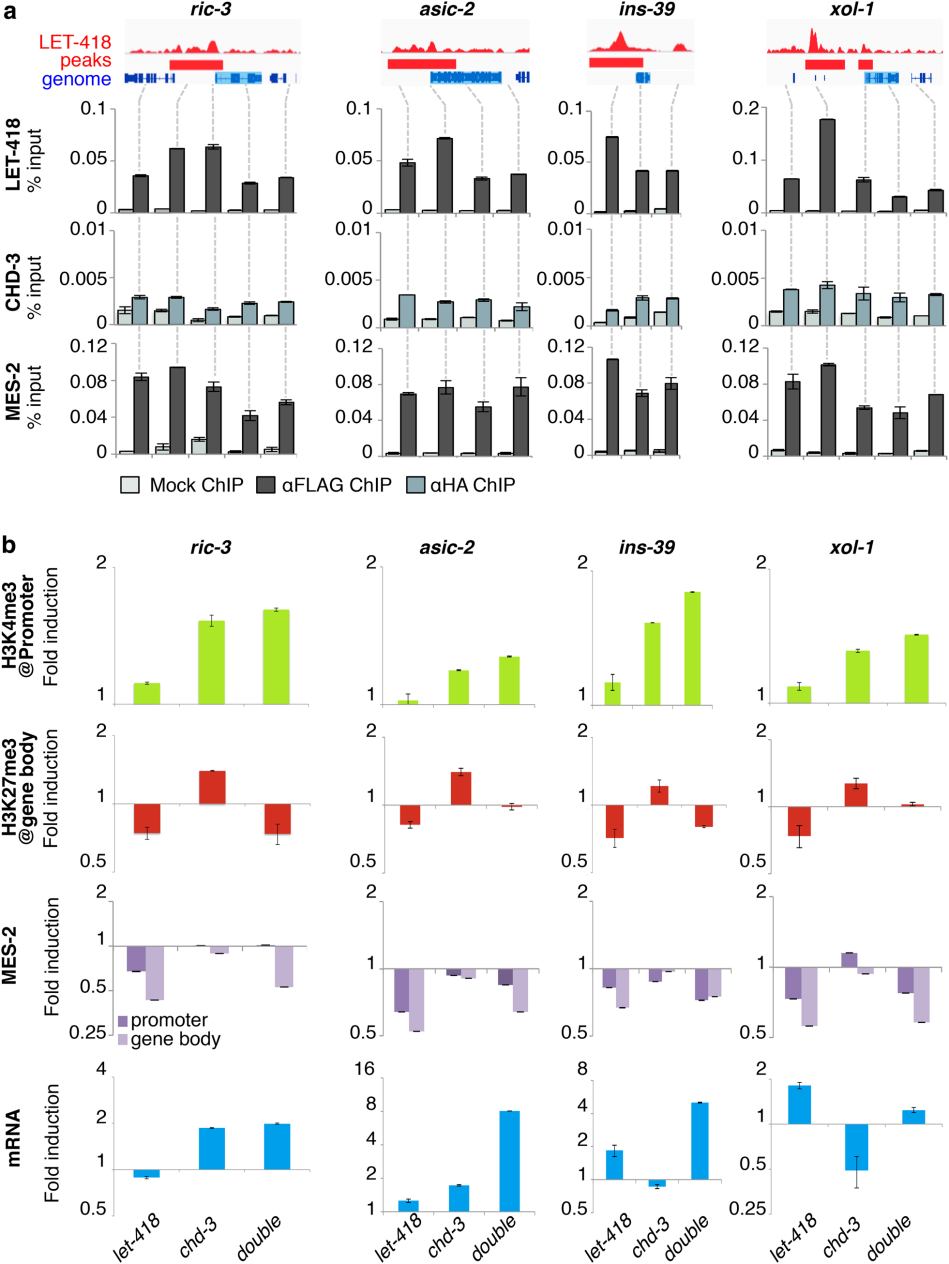
LET-418 and CHD-3 modulate histone marks and MES-2 recruitment at target genes. **a** ChIP-Seq validation of LET-418 binding patterns at four selected target genes by ChIP-qPCR in a wild-type genetic background. LET-418 ChIP-Seq profile (IGV browser—*red*) is represented at the top of the panel for the *ric*-*3, xol*-*1, asic*-*2*, and *ins*-*39* target genes (*blue boxes*). CHD-3::StrepIII::HA and MES-2::EGFP::3xFLAG enrichments were measured by ChIP-qPCR at these loci. Results are represented as % of the total DNA input added in each IP. *Error bars* qPCR measurement standard error. **b** ChIP-qPCR of H3K4me3, H3K27me3, and MES-2::EGFP::3xFLAG enrichment at target genes promoter and gene body, and mRNA expression levels of the same target genes, in the indicated genotypes. Results are represented as fold induction of the DNA recovery versus *control (RNAi)*-treated wild-type embryos, and for histone modifications only were normalized to total H3 levels, to obtain the histone mark enrichment independently of nucleosome concentration. mRNA levels of *asic*-*2, xol*-*1, ins*-*39*, and *ric*-*3* measured by qRT-PCR, represented as fold induction of mRNA expression versus *control(RNAi)*-treated wild-type embryos, and normalized to four housekeeping mRNA levels using the Best Keeper method. All ChIP-qPCR and qRT-PCR experiments were performed at least in duplicate, and one representative experiment is displayed here

We therefore decided to investigate the mechanisms of action of LET-418 and its homologue CHD-3 at the chromatin levels, by analyzing the effects of depleting these proteins, individually or simultaneously, on the chromatin content of the target genes they redundantly control. To identify such targets, we firstly cross-compared our RNA-Seq and ChIP-Seq data. Although the LET-418 ChIP-Seq was performed in mixed-stage embryos, we hypothesized that it was, at least partially, representative of LET-418 most enriched sites throughout development, and included genes regulated in the early developmental time points used in the RNA-Seq. Indeed, the gene overlap between the ChIP-Seq and RNA-Seq lists was small but significant in most cases, with LET-418 peak binding sites representing between 10 and 20 % of the genes deregulated in mutants (Additional file 1: Figure S3F and Table 3). Noticeably, most of the deregulated genes were enriched in LET-418 at their promoter (Additional file 1: Figure S3F), which is consistent with LET-418 being accumulated at pre-TSS regions of its principal target genes. A list of ≈300 candidate target genes redundantly and directly controlled by LET-418 and CHD-3 during early development was then built, by selecting the candidates which were more induced in double than in single mutants and physically enriched in LET-418 at their promoter. Since LET-418 promoter peaks are mostly located on genes involved in development, growth, and reproduction, we then picked from our list a few candidates with a characterized function in these processes. Their sequence was also checked for the absence of repetitive motifs to facilitate PCR analyses. Four representative target genes resulting from our selection are presented in this study: *ric*-*3* and *asic*-*2* (neuronal genes), *xol*-*1* (sex-determination gene), and *ins*-*39* (insulin-like peptide). Quantitative RT-PCR analysis (qRT-PCR) was used to verify their redundant control by *let*-*418* and *chd*-*3* (Fig. 5b). Similar to the RNA-Seq results, *ric*-*3, asic*-*2, and ins*-*39* were more deregulated in the double-mutant background compared to the single mutants, whereas *xol*-*1* was slightly less deregulated in the double mutant compared to what was observed in the single mutants (Fig. 5b, blue histograms). This discrepancy between the RNA-seq data (log2 fold change at the 100-cell stage in *let*-*418*: 1.76, *chd*-*3*: 0.78, *let*-*418;chd*-*3*: 2.9), and the validation is likely due the difference in the embryonic stages between the two experiments. ChIP-Seq trends were then validated by anti-FLAG ChIP followed by quantitative PCR (ChIP-qPCR) of mixed early embryonic populations on LET-418::3xFLAG in the *let*-*418(s1617)* null background (Fig. 1b), using primers located at strategic loci (Fig. 5a). Peak LET-418 binding measured by ChIP-qPCR correlated with peak ChIP-Seq signal at each target gene promoter (Fig. 5a). These results confirm that the selected genes are *bona fide* LET-418 direct targets in the embryo. Using anti-HA ChIP-qPCR against a CHD-3::Strep::HA construct in *chd*-*3 null* embryos, we measured the CHD-3 enrichment at LET-418 target genes (Fig. 5a). In spite of multiple tests with various anti-HA antibodies, chromatin recovered by anti-HA ChIP was only weakly enriched when compared with mock ChIP (Fig. 5a and data not shown), indicating that CHD-3 either did not bind to LET-418 target genes or had a weaker affinity for chromatin and was below the detection threshold of our assay.

Since LET-418 binds promoters with an active chromatin conformation, we asked whether depletion of the two Mi2 homologues had an effect on this chromatin environment by measuring the levels of the H3K4me3 and H3K27me3 modifications on our four candidates. Although all qPCR primers from Fig. 5a were checked during the assay, we chose to depict only the promoter loci for H3K4me3 levels and the ORF regions for H3K27me3 levels, as these marks accumulate preferentially at these respective loci (Fig. 4). Strikingly, we observed that depleting *let*-*418*, *chd*-*3*, or both Mi2 copies had distinct effects on H3K4me3 and H3K27me3 distributions, but these effects were the same for the four target genes analyzed. Generally, the ChIP-qPCR fold change relative to *control* worms was inferior to 2, indicating subtle, but reproducible, changes in the chromatin content at the studied loci (Fig. 5b).

H3K4me3 levels increased slightly or remained unchanged at the targets promoter in *let*-*418* single mutants, but was more markedly induced in *chd*-*3* and double *let*-*418; chd*-*3* mutants (Fig. 5b, green histograms), indicating that LET-418 and CHD-3 have a redundant function in repressing H3K4me3 levels at the promoter of target genes they redundantly control.

On the other hand, H3K27me3 levels were induced on the target ORF in *chd*-*3* mutants, but repressed or unchanged in *let*-*418* and double mutants (Fig. 5b, red histograms), suggesting that LET-418 was responsible for H3K27me3 deposition, whereas CHD-3 had the opposite function and restricted this deposition but only when LET-418 was present.

When compared with transcriptional changes occurring in the same embryonic samples, we globally observed a good link between histone mark content and transcriptional variation in *let*-*418* and *let*-*418; chd*-*3* mutants. The two neuronal genes, *ric*-*3* and *asic*-*2*, and the insulin peptide-coding gene *ins*-*39* showed correlated histone marks and transcriptional variations; most particularly, the highest transcriptional level was reached in double mutants, which displayed high H3K4me3 and low H3K27me3 marks and were therefore in the most “active” chromatin conformation (Fig. 5b).

However, discrepancies were generally observed in *chd*-*3* single mutants, which displayed high H3K27me3 and H3K4me3 levels compared to *control*, but for which transcription was not always repressed (Fig. 5b). Finally, since *xol*-*1* mRNA levels did not corroborate the RNA-Seq data, we could not draw any conclusions over this gene expression pattern (Fig. 5b). In both cases, we attribute these discrepancies to additional degrees of gene expression control, at either the chromatin or the transcriptional level, which we could not test in the present study.

Altogether, our data demonstrate that Mi2 proteins have differential roles in modulating H3K4me3 and H3K27me3 deposition at their common targets. LET-418, which binds preferentially H3K4me3-enriched and H3K27me3-depleted genes (Fig. 4), appears to restrict H3K4me3 deposition in a redundant manner with CHD-3 and to promote H3K27me3 deposition independently of CHD-3. Reversely, CHD-3 restricts H3K4me3 deposition redundantly with LET-418, and represses H3K27me3 deposition only in the presence of LET-418. Hence, we believe that the redundant repression of target genes by the two Mi2 proteins is the consequence of a fine-tuning between their partially overlapping and partially counteracting functions.

### MES-2/Polycomb recruitment to chromatin is differentially regulated by LET-418 and CHD-3

Surprisingly, we observed a direct link between LET-418 and H3K27me3 deposition at the chromatin of redundantly controlled target genes. Indeed, H3K27me3 marks decreased when LET-418 expression was reduced (Fig. 5b). This does not appear to be true for CHD-3, as single mutants actually display higher H3K27me3 levels in the presence of functional LET-418 (Fig. 5b). Our data suggest that LET-418 promotes the deposition of the negative H3K27me3 marks at its target genes, directly or indirectly, whereas CHD-3 acts as a modulator of this deposition. In *C. elegans*, H3K27 methylation is ensured by the Polycomb complex (PcG) and its three core components *mes*-*2,*-*3, and* -*6* [40]. To first determine whether the Mi2 proteins modulate Polycomb recruitment at their target genes, we performed ChIP-qPCR analysis of MES-2::3xFLAG::EGFP binding to *ric*-*3*, *asic*-*2*, *ins*-*39* and *xol*-*1* in single or double *let*-*418; chd*-*3* mutants, and compared them with control embryos (Fig. 5b, purple histograms). Our results correlated with the H3K27me3 ChIP-qPCR results in the same mutant backgrounds, since MES-2 was lost from promoter and ORF in *let*-*418* and *let*-*418; chd*-*3* mutants, consistent with H3K27me3 being lost in these mutants, but was retained in *chd*-*3* single mutants, in which H3K27me3 deposition increased (Fig. 5b). Altogether, our data suggest that LET-418 recruits MES-2 to its target genes, whereas CHD-3 functions as a repressor of this recruitment.

**Fig. 6:**
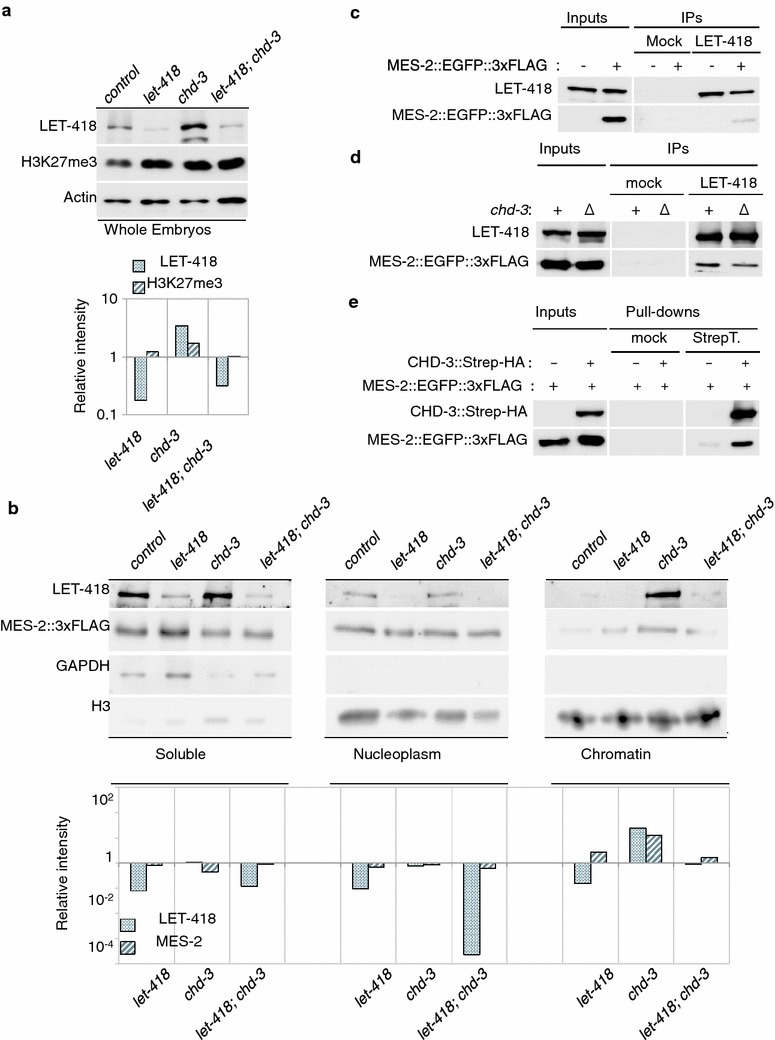
CHD-3 restricts the LET-418-dependent MES-2/Polycomb recruitment to chromatin. **a** Global levels of LET-418 and H3K27me3 proteins in whole embryonic extracts of wild-type or *chd*-*3(eh4)* null worms treated with *control*- or *let*-*418(RNAi)*. Upper panel: western blots; lower panel, quantification of the western blot, represented as relative intensity normalized to actin levels. **b** Subcellular fractionation experiment of soluble/cytosolic, nucleoplasmic, and chromatin fractions in embryos of the same genotypes as (**a**). LET-418, MES-2::EGFP::3xFLAG levels were assessed by western blotting, and fractionation was verified with proper cytosolic GAPDH versus nuclear total H3 separation (*upper panels*). *Lower panels* western blot quantification, normalized to GAPDH (soluble fraction) or to H3 levels (nucleoplasm and chromatin fractions). All fractions were loaded with equal protein amounts, and the selected western blots were exposed for the same duration. **c** Anti-LET-418 immunoprecipitation identifies MES-2::EGFP::3xFLAG as a LET-418 interactor in embryonic extracts. Western blots of LET-418 and MES-2::EGFP::3xFLAG in embryos expressing one or the two constructs (±) and immunoprecipitated for Mock (no antibody) or LET-418-binding proteins. **d** *chd*-*3* depletion does not disrupt the LET-418/MES-2 interaction. Anti-LET-418 immunoprecipitations performed in wild-type (+) or *chd*-*3(eh4)* (Δ) embryos and western-blotted for the indicated proteins. Mock and LET-418 IP western blots were selected at an identical exposure time. **e** Tagged CHD-3::StrepIII::HA interacts with MES-2::EGFP::3xFLAG in embryonic extracts. Strep-tactin pull-downs were performed in embryos expressing or not the CHD-3::strepIII::HA construct (±), and the presence of both tagged protein was detected by western blotting analysis. Mock IP: agarose resin only

To determine whether this mechanism was limited to a small number of genes, or was generally observable in the embryo, we investigated the effects of depleting Mi2 homologues on H3K27me3 levels and MES-2 levels and localization in embryonic extracts. Loss of *chd*-*3* led to a visible stabilization of LET-418, but also to a modest increase in H3K27me3 (Fig. 6a), which probably occurred post-transcriptionally since *let*-*418* and *mes*-*2* mRNA levels did not increase in this mutant (Additional file 1: Figure S5). Fractionation of the subcellular compartments showed that LET-418 protein levels, and to a lesser extent levels of a tagged version of MES-2, decreased in absence of functional *let*-*418,* but were stabilized on chromatin when *chd*-*3* was missing (Fig. 6b). To verify the affinity of LET-418 and CHD-3 for MES-2, we performed co-immunoprecipitation assays in embryonic extracts and determined that both LET-418 and CHD-3 could interact with MES-2 in vivo (Fig. 6c–e). Both proteins interacted with MES-2 and *chd*-*3* deletion did not influence the LET-418/MES-2 interaction (Fig. 6d), suggesting that CHD-3 might instead block the MES-2 access to chromatin, either by sequestering it outside of chromatin or by sterically blocking of the LET-418/MES-2 interaction platform, for instance. Altogether, these biochemical studies in embryonic extracts correlate with our ChIP-qPCR results and demonstrate that LET-418 is, at least partially, responsible for MES-2/Polycomb recruitment onto chromatin, whereas CHD-3 limits this recruitment. In addition our results suggest that the proposed mechanism of H3K27me3 modulation by LET-418 and CHD-3 might not be only restricted to the four target genes studied in this assay, but could concern a larger gene population.

## Discussion

Our study reveals that the *C. elegans* Mi2 homologues, LET-418 and CHD-3, though well conserved at the amino acid sequence level are both capable of distinct and common functions with regard to chromatin regulation.

### Mi2 proteins redundantly repress H3K4me3 deposition to specific target gene promoters

In this study, we demonstrate that a shared function for Mi2 homologues is to block H3K4 methylation at developmental gene promoters, which might be a requisite to limit their expression during the early stages of embryogenesis. The LET-418 positioning at a TSS-proximal locus might play a central role in this regulation. We also found that ASH-2, a member of the COMPASS complex responsible for H3K4me3 deposition, accumulated at this locus, suggesting a direct inhibition of COMPASS by LET-418-containing complexes. This hypothesis is in agreement with our previous model, in which LET-418 and the H3K4me3 demethylase SPR-5/LSD1 block COMPASS access to chromatin to maintain germ cells pluripotency and block somatic differentiation [42]. The observation that CHD-3 plays a redundant function in this mechanism suggests that the blockade of COMPASS activity at developmental gene promoters is of upmost importance to promote proper embryonic development and that a large epigenetic barrier complex might exist at the TSS-proximal site bound by LET-418. Considering that our transcriptomic analyses revealed multiple gene populations regulated by LET-418, CHD-3, or both, multiple tissue- and stage-specific epigenetic barriers against COMPASS might exist and include one or two Mi2 copies.

### A primordial Mi2 function in repressing the neuronal fate in early embryos

Strikingly, the redundant function of LET-418 and CHD-3 lies principally in repressing developmental genes in early embryos, among a predominant proportion of neuronal genes. Comparison of RNA-seq lists with the Spencer tissue-specific gene lists [22] was very efficient at demonstrating that absence of *chd*-*3* expanded the number of neuronal genes already up-regulated in *let*-*418* embryos, whereas CHD-3 itself did not repress neuronal genes. This underlines that the redundant function of LET-418 and CHD-3 occurs at neuronal genes physically targeted by LET-418. In addition, *let*-*418; chd*-*3* embryos precociously expressed larval-specific neuronal genes, suggesting that the LET-418/CHD-3 redundancy was essential to repress the neuronal fate in early embryos. This echoes the multiple reports of an essential role for NuRD in promoting a proper stem cell differentiation (reviewed in [41]) and confirms our previous finding that *let*-*418* repressed neuronal fates in germline cells [42], altogether suggesting that LET-418/NuRD complexes generally recruit chromatin and transcriptional machineries to neuronal genes.

### *C. elegans* Mi2 proteins oppositely regulate Polycomb recruitment to chromatin

A distinct function of the two Mi2 homologues lies in their relationships with the H3K27 methyltransferase Polycomb complex. Although both LET-418 and CHD-3 can interact with MES-2, LET-418 recruits Polycomb to a subset of its targets, whereas CHD-3 limits this recruitment. This could be performed by directly competing with LET-418 for the same targets, or by sequestering LET-418 and/or MES-2 outside of chromatin. A physical interaction between mouse CHD4 and the Polycomb subunit Ezh2 was already described and essential to block astrogenesis differentiation in the cortex [43]. This interaction was not proven in Embryonic Stem Cells (ESCs), but the NuRD histone deacetylation activity facilitated Polycomb access to chromatin [44].

In addition, considering the global increase of MES-2 on chromatin in *chd*-*3* mutants, and the large amount of down-regulated genes in *chd*-*3* single or *let*-*418; chd*-*3* double mutants only obeying to *chd*-*3* regulation, we believe that CHD-3 could also titer Polycomb out of chromatin for a specific gene population not targeted by LET-418. Interestingly, the down-regulated genes of *chd*-*3* mutants are specific to intestine- and pharynx-transcriptional programs, implying a function of the CHD-3/NuRD complex in specifying these organs. A synthetic pharynx developmental defect was already identified in *chd*-*3; pha*-*4* double mutants, supporting this hypothesis [45]. Nevertheless, we cannot exclude that CHD-3/NuRD also displays transcriptional activating functions. In adult human and murine hematopoietic cells, NuRD is recruited by and to FOG-1/GATA1 target gene and is essential to FOG-1-dependent transactivation [46, 47], indicating that NuRD can punctually potentiate transcription factor activity. Alternatively, one or a few major transcriptional repressors, themselves normally repressed by CHD-3, could be responsible for the *chd*-*3* down-regulated gene population, although we were not able to pinpoint any major transcriptional repressor in the up-regulated gene lists.

However, our favored model is that CHD-3 functions are necessary to limit LET-418-dependent H3K27 methylation at the promoter of important developmental genes, in order to avoid these genes to become fully silenced before the termination of differentiation.

### Mi2 homologues in higher eukaryotes: Duplication for enhanced functions in fine-tuning gene regulation?

Our studies demonstrate that two closely related homologues can have distinct target genes and molecular functions at the chromatin level, and provide a novel model for the molecular mechanisms of NuRD-dependent gene regulation during development. More than simple transcriptional repressors, we believe that Mi2 proteins, and their associated complexes, are fine-tuners of chromatin composition and transcription machinery accessibility. Although more studies will be necessary to identify the molecular differences between LET-418 and CHD-3 that led to such distinct functional outcomes, it becomes clear that the functions of both proteins are important enough to maintain both Mi2 copies in this organism. Considering the ubiquitous Mi2 duplications observed in the vertebrate subkingdom, we believe that a fine-tuning system of epigenetic control might have evolved in organisms with an ordered and complex embryonic development. The model we highlighted in *C. elegans* might therefore be transposable to vertebrate CHD3/Mi2α and CHD4/Mi2β, which are expressed simultaneously and nearly ubiquitously, and for which a functional distinction was not clearly identified. Indeed, similarly to *C. elegans* Mi2 copies, CHD3/Mi2α and CHD4/Mi2β are 71 % identical by BLASTP homology search, with central ATPase and Helicase domains being particularly well conserved. This close homology, added to their identical expression patterns, suggests subtle molecular differences, which could only be visible at the level of the genomic loci targeted by these proteins.

## Conclusion

Using a combination of transcriptomics and epigenomics analyses, we have shown that close Mi2 homologues have evolved both redundant and opposite functions in *C. elegans* to finely tune developmental gene expression in early development. Our observations provide a novel model, which could be applied to the study of detailed epigenetic mechanisms of developmental gene regulation in more complex organisms with similar duplications of core chromatin remodeling factors, in particular within the vertebrate kingdom.

## Methods

*C. elegans* strains, RNAi clones, oligonucleotides, and antibodies used in this study, as well as additional experimental details, are described in details in the Additional file 2: Additional methods.

### RNAi methods

RNAi experiments were performed by feeding as described [48], using HT115 bacteria expressing the indicated RNAi clones. Briefly, L4 mothers of the indicated genotype were fed on RNAi plates at 25 °C and allowed to lay fertilized embryos for 28 h. Their F1 progeny were then analyzed or harvested on the RNAi plates at the indicated time of growth, at 25 °C.

### RNA sequencing

Wild-type or *chd*-*3(eh4)* L4 hermaphrodites were fed for 24 h at 25 °C with HT115 bacteria expressing specific dsRNA against *gfp(control)* or *let*-*418*. Adult hermaphrodites were then dissected under a binocular. 2-cell stage embryos were isolated manually and incubated at 25 °C for 1 h (24-cell stage) or 3 h (100-cell stage) in M9 buffer. Approximately 200 embryos were collected for each sample. Total RNA was extracted with QIAzol (Qiagen, Germany) using 5 μg of linear polyacrylamide (GeneElute LPA, Sigma, MI, USA). Contaminating DNA was digested using DNase I (Qiagen), and RNA was purified using the RNeasy MinElute Cleanup kit (Qiagen). Quality and concentration of each sample were determined using the 2200 TapeStation and High Sensitivity RNA Screen Tape (Agilent, CA, USA). cDNA library preparation and RNA sequencing were performed at the Genomics Technology Facility (GTF) in Lausanne (https://www.unil.ch/gtf). Amplified cDNA was prepared from total RNA using the Ovation RNA-seq V2 system (NuGEN, CA, USA), and cDNA libraries were built using the TruSeq mRNA sample prep kit (Illumina, CA, USA). RNA sequencing (100 bp, single end) was performed on three biological replicates per sample with a HiSeq 2000 genome sequencer (Illumina). Four cDNA libraries were multiplexed per sequencing lane.

### RNAseq analysis

Raw reads (SE 100 bp) were obtained from GTF (UniL) and their quality checked with FastQC. (http://www.bioinformatics.babraham.ac.uk/projects/fastqc). Reads were remapped onto the reference genome Ce10 (WBcel215/WS220) with Tophat2/Bowtie2 [49] to obtain bam files. Read count by gene was obtained by HTSeq-count [50].

The DESeq [51] and EdgeR [52] were used in parallel to calculate the genes differentially expressed. Read counts were normalized by estimating the size factors and differential expression tested against the negative binomial distribution with Wald test and 10 % FDR via Benjamini–Hochberg correction. Comparisons were done pairwise (control vs. sample at 1 h and at 3 h, sample vs. sample at 1 h and at 3 h, and 1 vs. 3 h). The list of differentially expressed genes common to the two methods (significance threshold adj-*p* value <0.1) for each comparison was converted to an excel sheet, and processed lists of deregulated genes are provided in the Additional file 3: Additional Spreadsheet.

### Chromatin immunoprecipitation

Chromatin immunoprecipitation (ChIP) and quantitative PCR were performed following standard protocols and are described in details in the Additional file 2: Additional Methods.

### ChipSeq analysis

Reads (PE 100 bp) were obtained from the UniBe sequencing facility (Illumina HiSeq 2500) and their quality checked with FastQC. Reads were loaded in the BBCF HTSstation (http://htsstation.epfl.ch) [53]. Within BBCF, reads were remapped onto the reference genome Ce10 (WBcel215/WS220) using Bowtie2 and defaults options “–end-to-end –sensitive -k 20” [49] followed by additional post-processing steps [54]. The output was converted to BAM and filtered to keep only mapped reads with at most 5 hits in the reference. Peaks were called using MACS v1.4.0 [55] with the combined duplicate sample versus the input bam files and the following parameters “-s read_length -m 10,100 –bw = 200 –verbose 1 –keep-dup all.” The peak deconvolution algorithm of Rey et al. was applied. It analyses ChIP-seq signal within each MACS enriched region and produces a deconvolved density and a refined peak list [56]. Additional peaks were identified with the MACS v2 program [55] and merged to the previous file to complete the peak library. The BAM, BED and WIG files were downloaded for further use. BED files were annotated using BBCF scripts [53] and the annotation split per gene using a home made perl script. The nearest feature was used with promoter region defined as 2000 bp upstream of a gene and intergenic region defined as 20,000 bp between genes. See http://bbcf.epfl.ch/bbcflib/bbcflib_gfminer.html#bbcflib.gfminer.stream.annotate.getNearestFeature for more details. Splitting was required when several features are found for a peak. Scripts are available upon request. Genome-wide LET-418 binding profiles were visualized and aligned to the *C. elegans* genome version ce10 using the Integrated Genome Viewer (IGV) [57, 58]. Datasets downloaded from the ModEncode consortium website are listed in the Additional file 2: Additional Methods.

### Protein biochemistry

Cellular compartment fractionation, immunoprecipitations (IPs) and western blotting analysis were performed as described elsewhere [42, 59] with minor modifications detailed in the Additional file 2: Additional Methods.

## Additional files

10.1186/s13072-016-0091-3 LET-418 and CHD-3 are well-conserved proteins with distinct and redundant functions in *C.elegans* embryonic development. **Figure S2:** Supplemental analysis and validation of RNA-Seq experiment. **Figure S3:** ChIP-sequencing analysis and RNAi screen to identify genes targeted by the LET-418/CHD3 redundancy. **Figure S4:** LET-418 is depleted at H3K27me3-enriched loci. **Figure S5:** *mes-2* mRNA expression is not perturbed by Mi2 depletions. **Additional Table 1:** The LET-418::3xFLAG transgene fully rescues the developmental arrest of *let-418* mutants. **Additional Table 2:** LET-418 associates with genes involved in growth, development, and sex differentiation. **Additional Table 3:** Cross-comparison between LET-418::3xFLAG ChIP-Seq and Mi2 mutant RNA-Seq experiments.
10.1186/s13072-016-0091-3 RNA-Seq deregulated genes. Processed lists of deregulated genes in the indicated genotypes versus control are displayed below. A threshold of 1.5 log2 fold change and a *p* value <10 % were applied. *let*-*418*: *wild*-*type; let*-*418(RNAi)*-treated embryos; *chd*-*3*: *chd*-*3(eh4); controlGFP(RNAi)*-treated embryos; *chd*-*3_let*-*418*: *chd*-*3(eh4); let*-*418(RNAi)*-treated embryos. All fold changes are calculated versus *wild*-*type; control(RNAi)*-treated embryos. _1 h and _3 h correspond to the 24- and 100-cell stages, respectively.
10.1186/s13072-016-0091-3 Supplementary Methods.

## Abbreviations

TSS: transcription start site
ORF: open reading frame
PcG: Polycomb group
NuRD: nucleosome remodeling and deacetylase
HDAC: histone deacetylase complex
MBD: methyl binding domain
MTA: metastasis tumor antigen
CHD: chromatin and helicase domain
PHD: plant homology domain
ChIP: chromatin immunoprecipitation
NDR: nucleosome depletion region
GO: gene ontology
HA: hemagglutinin
ESC: embryonic stem cell
