# Supplementary material for: Fine-tuning of chromatin composition and Polycomb recruitment by two Mi2 homologues during C. elegans early embryonic development
Source: Epigenetics Chromatin. 2016 Sep 15;9:39. doi: 10.1186/s13072-016-0091-3 (PMC5024519; doi:10.1186/s13072-016-0091-3)
Supplement: Supplementary file 1 — 10.1186/s13072-016-0091-3 LET-418 and CHD-3 are well-conserved proteins with distinct and redundant functions in C.elegans embryonic development. Figure S2: Supplemental analysis and validation of RNA-Seq experiment. Figure S3: ChIP-sequencing analysis and RNAi screen to identify genes targeted by the LET-418/CHD3 redundancy. Figure S4: LET-418 is depleted at H3K27me3-enriched loci. Figure S5: mes-2 mRNA expression is not perturbed by Mi2 depletions. Additional Table 1: The LET-418::3xFLAG transgene fully rescues the developmental arrest of let-418 mutants. Additional Table 2: LET-418 associates with genes involved in growth, development, and sex differentiation. Additional Table 3: Cross-comparison between LET-418::3xFLAG ChIP-Seq and Mi2 mutant RNA-Seq experiments. [file 13072_2016_91_MOESM1_ESM.pdf]

Additional Figures

Fine-tuning of chromatin composition and Polycomb recruitment by two Mi2 homologues during *C. elegans* early embryonic development

Stéphanie Käser-Pébernard, Catherine Pfefferli, Caroline Aschinger, Chantal Wicky

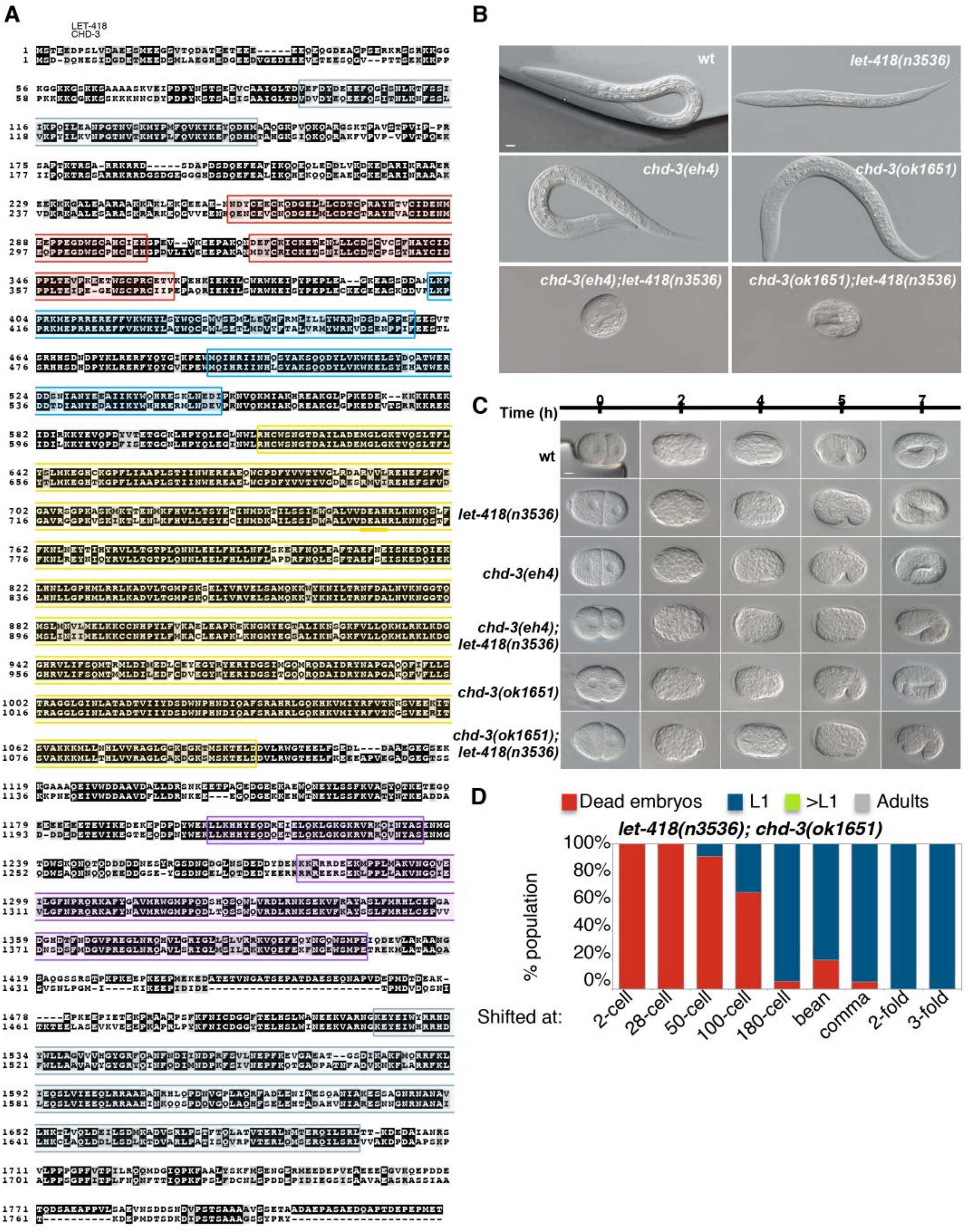

Figure S1: LET-418 and CHD-3 are well-conserved proteins with distinct and redundant functions in *C.elegans* embryonic development. (A), Protein alignment of LET-418 and CHD-3. Black boxes: amino acid identity, grey boxes: sequence similarity. Colored boxes delimitate the protein domains defined in figure 1B. (B) and (C), Nomarski images of *control*, or *chd-3(ok1651)* worms in *wild-type* or temperature-sensitive *let-418(n3536)* background, shifted at 25°C during 24h post egg laying (C) or at

the indicated time points (D), parallel the data observed with the other *chd-3(eh4)* null mutant. Scale bar: 10µm. (D) Redundant functions of LET-418/CHD-3 are required before the 100-cell stage. *let-418(n3536); chd-3(ok1651)* embryos were shifted at 25°C to inactivate LET-418 activity at the indicated embryonic stages, and analyzed for the resulting phenotypes. Between 19 and 33 embryos were tested per condition. Data in *chd-3(ok1651)* worms confirm the ones obtained with *chd-3(eh4)* null mutants.

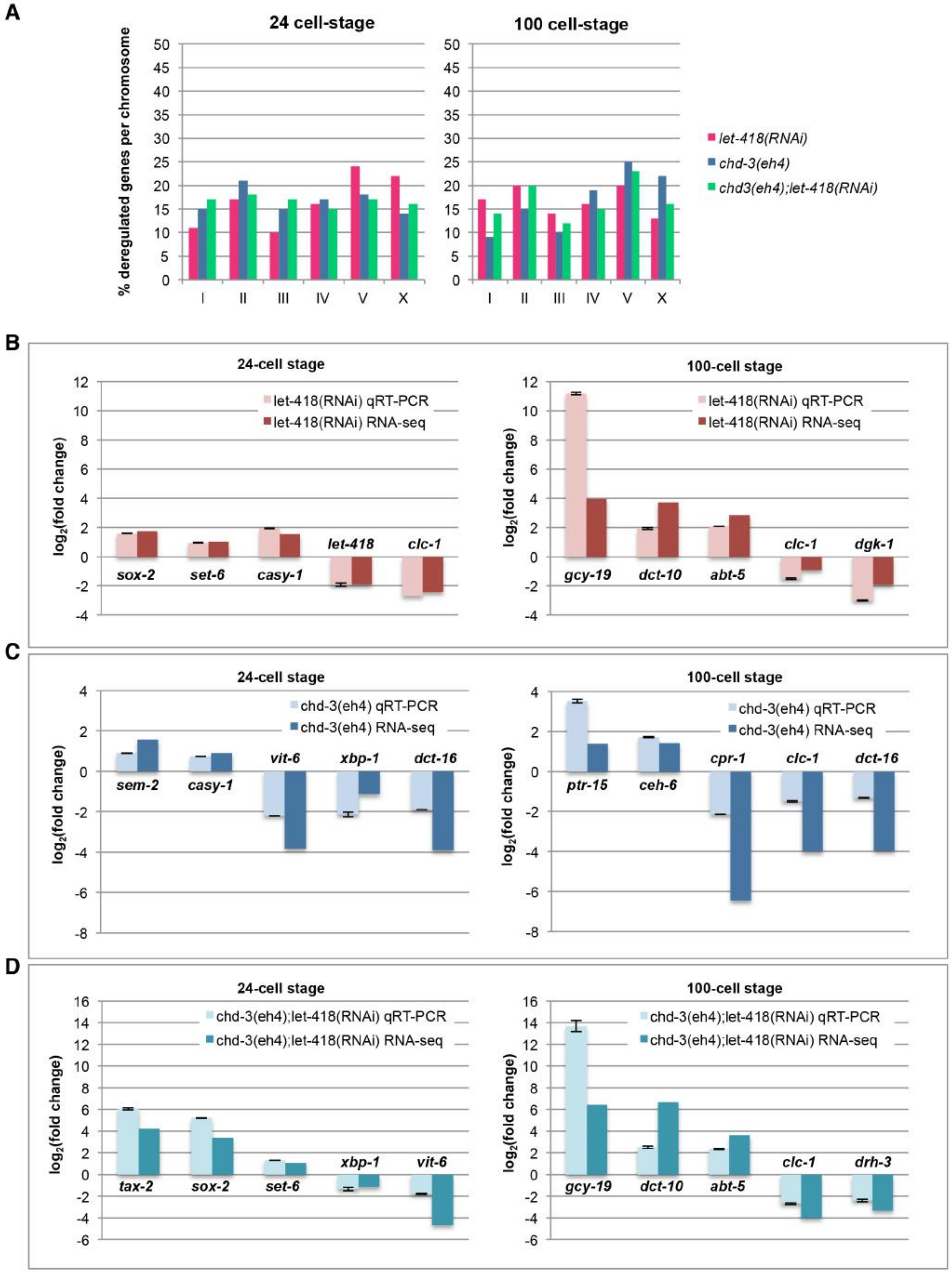

Figure S2: Supplemental analysis and validation of RNA-Seq experiment. (A), distribution of de-regulated genes on the six *C.elegans* chromosomes. Percentage of de-regulated genes in *wild-type; let-418(RNAi)*, *chd-3(eh4)*; *control(RNAi)* and *chd-3(eh4); let-418(RNAi)*-treated vs *wild-type; control(RNAi)*-treated embryos, per chromosome. (B-D), Validation of RNA-sequencing results by qRT-PCR. Comparison between quantitative RT-PCR and RNA-sequencing expression data for selected genes in *wild-type; let-418(RNAi)* (B), *chd-3(eh4); control(RNAi)* (C), and *chd-3(eh4); let-418(RNAi)* (D) embryos relative to *wild-type; control(RNAi)*-treated embryos, at 24- and 100-cell stages. Relative mRNA expression levels determined by qRT-PCR were normalized to tbb-1 levels using the $2^{\Delta Ct}$ method and data were represented as the Log2 fold change relative to control embryos. Error bars: standard error of the mean. Genes were de-regulated in the same direction (up or down), although the level of de-regulation was not always identical between the RNA-Seq and qRT-PCR experiments.

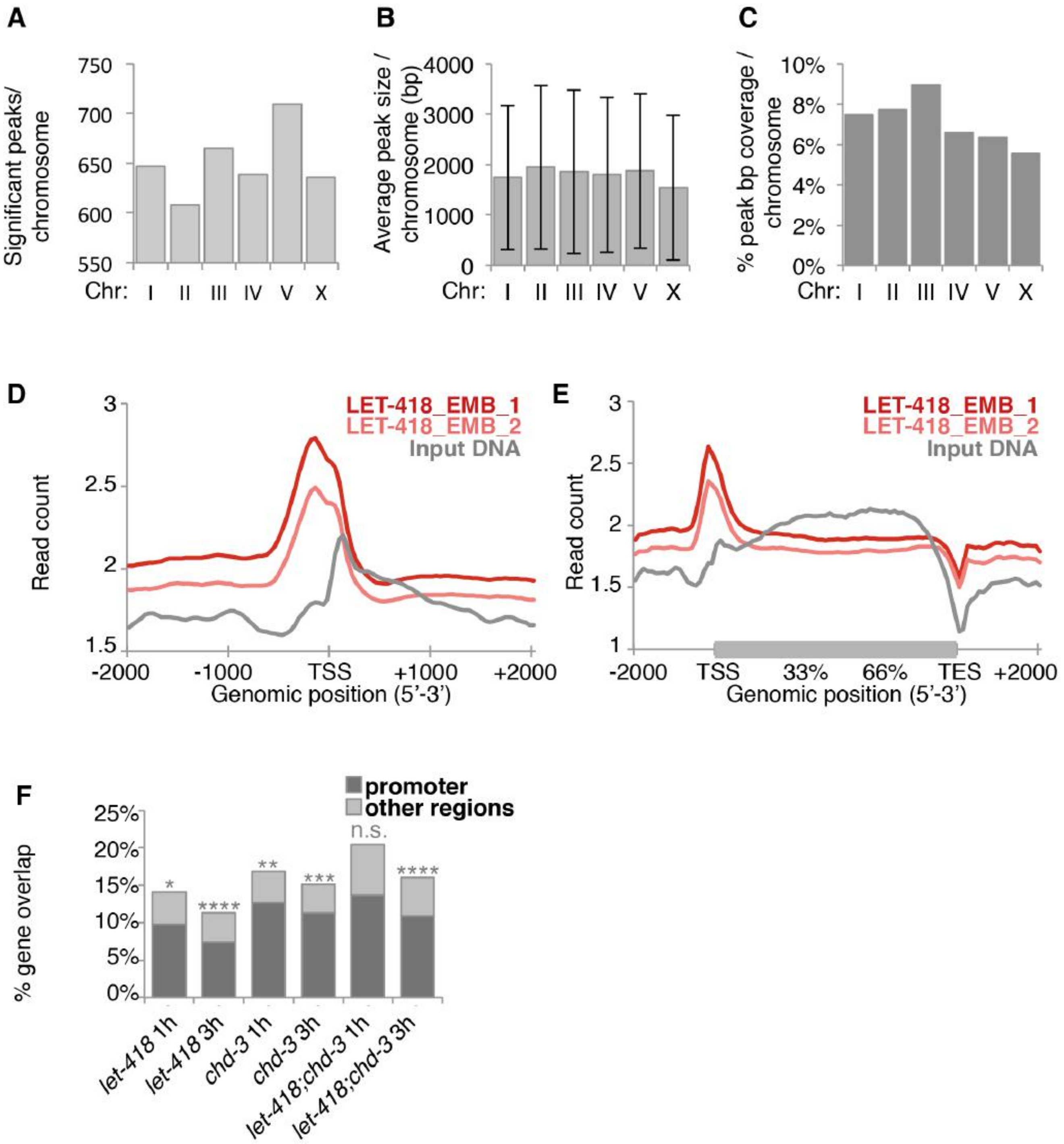

Figure S3: ChIP-sequencing analysis identify genes targeted by the LET-418/CHD3 redundancy. (A), distribution of LET-418::3xFLAG significant ChIP-Seq peaks per *C.elegans* chromosome. (B), average peak size per chromosome (bp). (C), percentage of the chromosome size covered by the LET-418 most significant peaks. (D-E), ngsplot of LET-418::3xFLAG duplicate ChIP-Seq experiments (red lines) compared with non-enriched input DNA sequencing, relative to TSS (D) or genebody (E) position. (F), gene overlap between de-regulated genes of the indicated RNA-seq samples and directly bound LET-418::3xFLAG ChIP-Seq target genes, and proportion of genes bound by LET-418 in their promoter or other regions.

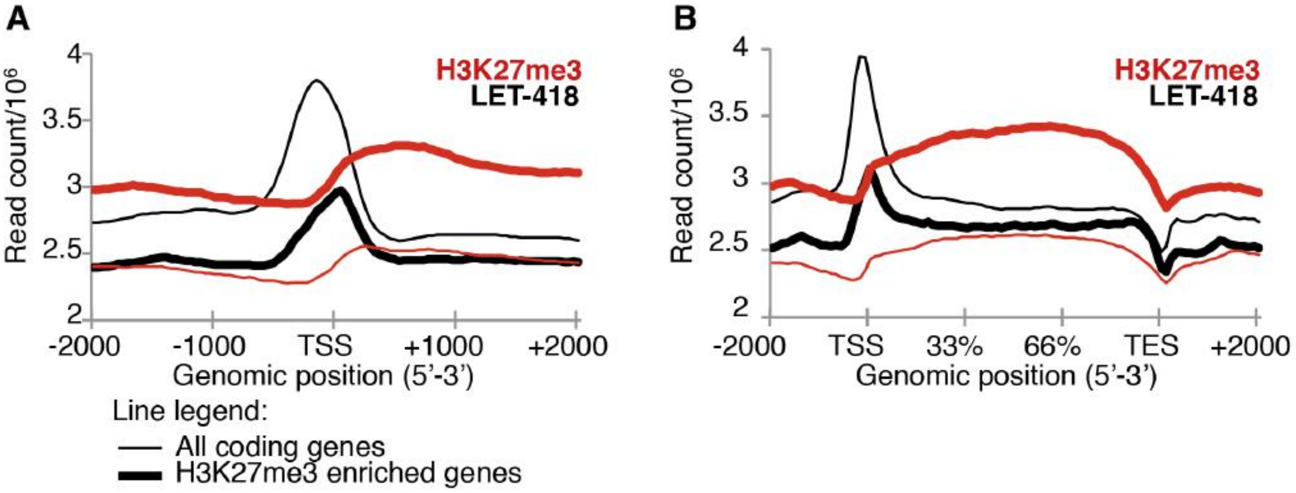

Figure S4: LET-418 is depleted at H3K27me3-enriched loci. (A-B), ngsplot of LET-418 and H3K27me3 at all coding genes or at genes enriched for H3K27me3 marks, relative to TSS (A) or gene body (B) position. Results are represented as read counts per million mapped reads.

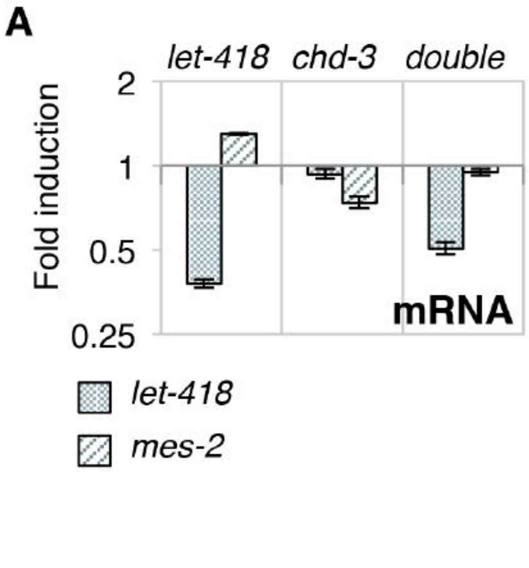

Figure S5: *mes-2* mRNA expression is not perturbed by Mi2 depletions.
A mRNA levels of *-418* and *mes-2* in *wild-type; let-418(RNAi)*, *chd-3(eh4); control(RNAi)*, and *chd-3(eh4); let-418(RNAi)*-treated embryos, measured by qRT-PCR, represented as fold induction of mRNA expression vs *control(RNAi)*-treated wild-type embryos, and normalized to four housekeeping mRNA levels using the Best Keeper method. All qRT-PCR experiments were performed at least in duplicate, and one representative experiment is displayed here.

Genotype	% dead embryos	% L1 larvae	% > L1 larvae	n=
let-418(n3536ts)	6.0	94	0.0	781
*[LET-418::3xFLAG];let-418(n3635ts)**	2.5	0.0	97.5	1220
wt	1.5	0.0	98.5	958

Additional Table 1: The LET-418::3xFLAG transgene fully rescues the developmental arrest of *let-418* mutants. The progeny of 5 to 7 worms of the indicated genotype grown at 25°C was scored for L1 arrested larvae.*The complete genotype of this strain (FR1355) is indicated in the Material and methods section.

GO term categories, clustered	LET-418::3xFLAG
**Growth regulation**	22.64%
**Post-embryonic/larval development**	19.38%
**Reproductive processes, genitalia/sex differentiation**	10.26%

Additional Table 2: LET-418 associates with genes involved in growth, development, and sex differentiation. GO term association of annotated genes for most significant LET-418::3xFLAG (this study) and MEP-1::EGFP::3xFLAG (ModEncode) binding regions in embryonic extracts. DAVID GO term search results were clustered and the three most important categories are listed here. P values were all significant (<0.01).

RNA-Seq sample	# de-regulated genes	# LET-418 binding sites (n=5118)	pvalue
let-418(RNAi) vs wt; control(RNAi), 24-cell	235	33	0.023 (*)
let-418(RNAi) vs wt; control(RNAi), 100-cell	434	49	4.03e-07(****)
chd-3(eh4); control(RNAi) vs control(RNAi), 24-cell	879	148	0.0064(**)
chd-3(eh4); control(RNAi) vs control(RNAi), 100-cell	695	105	0.00028(***)
chd-3(eh4); let-418(RNAi) vs control(RNAi), 24-cell	1611	330	n.s.
chd-3(eh4); let-418(RNAi) vs control(RNAi), 100-cell	1371	220	1.93e-05(****)

Additional Table 3: Cross comparison between LET-418::3xFLAG ChIP-Seq and Mi2 mutant RNA-Seq experiments. Intercepts were significant, to the exception of the *chd-3(eh4); let-418(RNAi)* sample which was not significantly enriched in LET-418 direct binding sites (n.s.).
