# Supplementary material for: Fine-tuning of chromatin composition and Polycomb recruitment by two Mi2 homologues during C. elegans early embryonic development
Source: Epigenetics Chromatin. 2016 Sep 15;9:39. doi: 10.1186/s13072-016-0091-3 (PMC5024519; doi:10.1186/s13072-016-0091-3)
Supplement: Supplementary file 3 — 10.1186/s13072-016-0091-3 Supplementary Methods. [file 13072_2016_91_MOESM3_ESM.docx]

**Additional Methods**

**Fine-tuning of chromatin composition and Polycomb recruitment by two Mi2 homologues during *C. elegans* early embryonic development**

**Stéphanie Käser-Pébernard, Catherine Pfefferli, Caroline Aschinger, Chantal Wicky**

**Additional Methods**

**C.elegans strains and culture**

Worm culture was performed under standard culture conditions [1], unless otherwise stated.

The following strains were used: N2; FR843: *let-418(n3536ts)V;* FR1122: *let-418 (n3536ts) V; chd-3(ok1651) X;* FR1156: *chd-3(eh4)X*; FR1158: *chd-3(ok1651)X;* FR1213*: let-418(n3536);chd-3(eh4) X*; FR1263: *unc-119(ed3)? III; SwSi2[cb-unc-119(wt);chd-3p::chd-3ORF::Strep-HA::tubulin3'UTR(II)]; chd-3(eh4) X;* FR1355: *SwSi3[cb-unc-119(wt); let-418p::let-418cds:3xFLAG::let-418-3’UTR II], unc-119(ed3)III (?), let-418(n3536ts)V*; FR1358: *SwSi3[cb-unc-119(wt); let-418p::let-418cds:3xFLAG::let-418-3’UTR II], unc-119(ed3)III (?), unc-46(e177) let-418(s1617)V*; FR1466: *unc-119(ed3) III; chd-3(eh4) X, ddIs262 [mes-2::TY1::EGFP::3xFLAG(92C12) + unc-119(+)]*; FR1467*: unc-119(ed3) III; SwSi2[cb-unc-119(wt);chd-3p::chd-3ORF::Strep-HA::tubulin3'UTR(II)]; chd-3(eh4) X, ddIs262 [mes-2::TY1::EGFP::3xFLAG(92C12) + unc-119(+)]*; TH458: *unc-119(ed3)III; ddIs262[mes-2::TY1::EGFP::3xFLAG(92C12) +unc-119(+)]*.

**Phylogenetic tree, domain conservation and protein alignments**

Whole protein sequences from the indicated species were obtained from the UNIPROT database [2] and aligned with MUSCLE [3]. The MUSCLE-generated sequence alignment was then analyzed via the iTOL tool [4] to generate an unrooted phylogenetic tree.

Domain identification and conservation of the LET-418 and CHD-3 *C.elegans* proteins was assessed using data available in the Conserved Domain Database [5].

To generate a graphic LET-418/CHD-3 protein alignment, a CLUSTAL alignment file was first generated using the two protein sequences. This alignment was then processed via the BOXSHADE server (http://www.ch.embnet.org/software/BOX_form.html) to identify blocks of identities or similarities between the two proteins.

**RNAi constructs**

RNAi constructs were all amplified from the Ahringer RNAi library [6], except for the control RNAi control (pPD129.36 (empty vector) or pPD128.110 (GFP RNAi control vector), Fire lab L4440), *let-418*, and *mep-1* RNAi vectors (pFG98 and pMP167 RNAi vectors; information available upon request).

**Chromatin Immunoprecipitation**

Chromatin Immunoprecipitation (ChIP) was performed as follows. Early embryonic populations of the indicated genotypes were harvested from synchronized mothers by bleaching, collected and washed, cross-linked in 2% formaldehyde for 30’ at room temperature, then the cross-linking reaction was quenched with 125mM Glycine for 20’ at room temperature. Collected embryos were washed, snap-frozen in liquid nitrogen, and sonicated directly in 1ml FA-150 ChIP lysis buffer (50mM HEPES pH7.5, 1mM EDTA pH8, 1% Triton-X-100, 0.1% Sodium deoxycholate, 150mM NaCl, 1mM DTT, 0.2mM PMSF, Complete protease inhibitors (Sigma-Aldrich, MI, USA) with a Misonix sonicator 3000 with a microprobe, output 5.0, 8-10 cycles of 15’’ per pellet, on ice. Sonication efficiency (ladder size from 300 to 800bp) was checked before proceeding. Soluble lysates were recovered by centrifugation, and proteins quantified using the Bradford assay (Bio-Rad, CA, USA). 1 to 1.5mg lysate was used per IP. To decrease background, lysates were pre-cleared with 20ul naked Protein G dynabeads (Life technologies, CA, USA). 1% pre-cleared lysate were put aside as INPUT DNA, and de-crosslinked in parallel to ChIP samples without any ChIP enrichment. To perform the IP, 50ul Protein G Dynabeads were pre-adsorbed with 4-8ug of the indicated antibodies (see below), prior to be mixed with the pre-cleared protein lysates for an overnight incubation at 4°C. IPs were then washed sequentially with 1ml of the following buffers: 2 washes with FA-150, 2 washes with FA-500 (same recipe as FA-150 but with 500mM NaCl), 1 wash with FA-1000 (same recipe as FA-150 but with 1M NaCl), 1 wash with DOC (250mM LiCl, 1% NP-40, 1% sodium deoxycholate, 1mM EDTA Ph8, 10mM Tris-HCl pH8), 2 washes with 1xTE (10mM Tris-HCl pH8, 1mM EDTA). Beads were then re-suspended in 150ul TES (1xTE, 1% SDS), vortexed, and heated at 75°C 15’ to detached complexes from beads. Input DNAs were also diluted in 150ul TES at this stage. Reverse cross-linking was performed overnight at 65°C in the presence of 2ul Proteinase K (10mg/ml, Sigma-aldrich). Putative RNA contaminants were eliminated by a 30’ RNase A treatment. The enriched chromatin was then purified using the Qiaquick PCR purification kit (Qiagen, CA, USA) and eluted in 200ul ddH20. Volumes were then increased up to 400ul for quantitative PCR assays, or sent directly for quality analysis to the Bern Next Generation Sequencing platform (<http://www.ngs.unibe.ch>).

The following antibodies were used in this study: anti-FLAG M2 (Sigma-aldrich); anti-HA.11 (Biolegend, CA, USA, formerly Covance #MMS-101P); anti-H3K4me3 # 07-473 and anti-H3K27me3 # 07-449 (Merck-millipore, MA, USA); anti-H3K36me3 #9050 and anti-total H3 #1791 (Abcam, UK).

**Quantitative PCR (qPCR)**

ChIP sample analysis by qPCR was performed with 3ul ChIP sample per well in duplicate, using the indicated primer pairs and KAPA SYBR Fast qPCR 2x mix (KAPA biosystems, MA, USA), on a Rotor-gene 6000 thermocycler (Qiagen), according to KAPA SYBR Fast mix manufacturer’s instructions. For anti-FLAG ChIP, data are represented as the % DNA recovery (% input) in ChIP samples relative to the initial amount of DNA in the inputs. For ChIP sample enrichment in histone modifications, % DNA recovery in the histone mark ChIP samples were normalized to the % DNA recovery in the total histone H3 ChIP sample of the same genotype, and data are finally represented as the fold induction/repression in *let-418*, *chd-3*, or *let-418;chd-3* mutants compared to control embryos. Error bars represent the standard deviation of the duplicate measurements. Each experiment has been performed as distinct biological du- or tri-plicates, but only one representative experiment is shown.

The following primers were used for ChIP-qPCR analyses:

*ric-3* upstream: F TGGAGCAAGGAACCAGATAA, R CAGTTTGCATGGACTCACCT

*ric-3* promoter distal: F GTGGCCTAGGAAACTCTTGC, R TCCAAACGTAAGTTATGAGGCTT

*ric-3* promoter proximal: F GGAGCTGCTCTGAGCTCTTT, R CGAACTATTGACAGAGAAATGGA

*ric-3* gene body: F TGCAGAAGATGCTGAAGAAGA, R TTTAGGTCTCCGCCTTCG

*ric-3* downstream: F TGCGCACTGTACAGAAGTGA, R TATCTCACTCTCGGGTGTCG

*xol-1* upstream: F CGCACTTCCAGATAACTCCA, R GAAACTACGCCGAAGGAAGT

*xol-1* promoter distal: F GCCAGTTGATGGTGGTATGA, R AAAGAGGCTGTTTGCCATCT

*xol-1* promoter proximal: F TGTAAGACCACACACGACGA, R GAGGACGCAGACACGTTAGA

*xol-1* gene body: F TGCATATTTGATCGAATGCC, R CCTGGGCCTAATTTGAGAAG

*xol-1* downstream: F TGAGGATTGCATATTCTGGTG, R ATGGCGGCCAATAGTCATA

*asic-2* promoter distal: F AGAGCAACCACGTCAAACTG, R AATTTGCGATCGCCTCTC

*asic-2* promoter proximal: F TGGAACAGAAACACACAACAAA, R CCACGTGTGGTACAACTGCT

*asic-2* gene body: F AACGTTCGAGCAAAGAAACC, R TCCAAACCTTGTAATAATCCGA

*asic-2* downstream: F CCAGAAACGAATTGAACAAAGT, R CTCCAAATGATCGATTTCCTT

*ins-39* promoter: F TTTCCGAATCTCGATTTCTTG, R ATCCTAATTTGGCGGAAGG

*ins-39* gene body: F GAAGACTTCCTGGAGTTCCG, R GAGCAAATGACTGCCAAGAA

*ins-39* downstream: F CCTATCCCATCTCTTCAGGTG, R ATGGTGGGAGCTAGGTCAAT

**qRT-PCR**

For mRNA expression analyses of RNA-Seq validation, amplified cDNAs were prepared from total RNA (see extraction method in RNA-Seq chapter above) using the ovation RNA-seq system V2 (NuGEN). In mRNA expression analyses of mixed embryonic populations, embryonic pellets corresponding to the ChIP experiment (but not cross-linked) were treated for RNA extraction using the RNeasy mini kit (Qiagen). Quantitative real-time PCR was performed in triplicates using the SensiFast SYBR No-ROX Kit (Bioline) or the KAPA SYBR Fast kit (KAPA biosystems). PCR reactions were run in the Corbett Rotor-Gene 6000 thermocycler according to PCR mix manufacturer’s instructions. Primers were specifically designed to span exon-exon junctions. Relative expression levels were normalized to the *tbb-1* level in the RNA-Seq validation experiment, or for experimental mRNA measurements to the Best Keeper Ct value calculated between 4 four different housekeeping genes (*act-1, tbb-1, 18s* and *rpc-1* [7]), using the 2^-∆∆Ct^ method. Results are represented as the fold induction between test (mutants) and control samples. The following primer pairs were used in this study: (all sequences are 5’-3’, F= Forward and R= Reverse).

*sox-2* : F ATGAAGCGAAACGACTACGC, R ATGAAGCGAAACGACTACGC

*set-6*: F AGGATTGCGGACTTTCGTC, R GCATTAGACGGATGTCGTTG

*tax-2*: F CCAAGACTGTGATCGTGCTC, R TCCAACGTCTCCCTTCAAAC

*casy-1*: F ACAGAGTGACGGAGGAATGC, R CACTTTCTCCATCGGTTTCC

*vit-6*: F AGCAAGTCCGCAATGAGAAG, R ACCTTGTTCTCGGTCCATTG

*xbp-1*: F TCCGTCGTATGGATTCCAAG, R GGGCTCTTGAGATGTTCGAG

*dct-16*: F  GTGATGCTGCCACTTCTTTG, R AGCTCGGCCTTCTTCTTCTC

*gcy-19*: F AACAAGGCCAAACTGTGGAG, R TGACAACCTGAAACGGTGAG

*dct-10* : F GTCGGGTTCCTCAGTCAAAC, R TCCCAACGAGTTGATGACAG

*abt-5*: F TGGTACACCGACAAGTGCTC, R TGAATATTGCTGCCACAAGC

*clc-1*: F CTCTCAGGGAACCCAGTGTC, R AGGCAAGGCATGTCAAAAAG

*drh-3*: F ATTGTTGCTCCGACTGGTTC, R GCACAAGGAGCACAACTCTG

*let-418*: F AGCTCCGCCAGTTCTTAGTG, R GGTTCTGGCTCATCTGTTGG

*dgk-1*: F AAGGAGGAGGGCAACTTTTC, R CGGATACTTCCACCTTGAGC

*sem-2*: F TCCCCTCAGAATGACGAATC, R CTGATGGTGGTGAAGTGTGC

*ptr-15*: F TGACGATTCTCCACTTGTTCC, R GCATTCTGTCCACCATTTCC

*ceh-6*: F AGGTTGTCCGTGTCTGGTTC, R TAATGATTGACGACGGCTTG

*cpr-1*: F AGCTGGATGCAAACCATACC, R AGACTCCGAAGTGCTTGTCC

*asic-2*: F CTTACGGTCACGCAAACAAA, R CCCACGTGAGTTTACTCCAA

*ric-3*: F GATGCTGCCGATAAGAGGTC, R TCTTTCAGTGCAACCTCAAGAT

*xol-1*: F TTCGAGCTGCAAATTCCAT, R CGTTGTCCCGTTTGAGTTT

*ins-39*: F TGGCTATCTACTTGAACATCAG, R TCTGCATTGCCTCCATGTTC

*mes-2*: F CAGTGCTACACGAAGGCTTG, R CGTCGCATTTGCACATATTA

*tbb-1*: F TCCATGAGAGAGGTTGACGA, R TCGCACACAGCAGTCTTGACG

*rpc-1*: F GACGTGATTCATGGAGTTTCG, R GGCTTTCTTGTCGTATTTCTGC

*18s*: F AGTAGCAAGGAGAGGGCAAG, R TTAACCGCAGCAATAACGAG

*act-1*: F ATCCATTGTCGGAAGACCAC, R GTAAGGATACCTCTCTTGGATTGG

**ModEncode datasets**

ModEncode datasets were downloaded from [http://www.modencode.org](http://www.modencode.org/) [8] and annotated similarly with BBCF scripts. The following ModEncode files were used in this study: Embryonic ChIP-Seq samples (#experiment reference): H3K4me1(#5158), H3K4me2(#5157), H3K4me3(#5166), H3K36me1(#5167), H3K36me2(#5164), H3K36me3(#5165), H3K79me1(#5168), H3K79me2(#5169), H3K79me3(#5170), H3K27ac(#5159), H3K27me1(#5160), H3K27me3(#5163), H3K9AcS10P(#5149), H3K9me1(#5171), H3K9me2(#5161), H3K9me3(#5170), H4K20me1(#6210), ASH-2(#6291), HTZ-1(#6218); Young adult ChIP-Seq samples: germline-enriched CHD-3(#5207). The average profiles graphics of genomic positions relative to TSS/TES were generated from the bam files by ngsplot [9].

**Cellular compartment fractionation**

Embryonic pellets of the indicated genotypes were treated for subcellular fractionation as described elsewhere [10], using the Qproteome cell compartment kit (Qiagen) with modifications. Assays were performed at 4°C and all buffers were complemented with protease inhibitors. Briefly, embryos were resuspended in 200ul CE1 buffer and disrupted by glass bead beating. Broken embryos were cleaned-up three times through Qiashredder columns (Qiagen). Lysates were centrifuged 10’ at 500g and supernatants, representing the soluble fractions, were collected. Pellets were treated with 200ul CE2 buffer 30’ at 4°C, spun for 10’ at 3’000g, and supernatants, representing the membrane fractions, were discarded. To separate nucleoplasm from chromatin, pellets were then first treated with 100ul CE3 buffer, incubated 30’ at 4°C, and centrifuged 10’ at 5’000g. Supernatants, representing the nucleoplasm fraction, were collected. The chromatin contained in the pellets was then digested with benzonase, 15’ at room temperature, and 100ul CE3 buffer were added before high-speed centrifuging 10’ at 16’000g. Supernatants, containing released chromatin, were collected, whereas the insoluble pellets were discarded. All fractions were precipitated with ice-cold ethanol, air-dried, and resuspended in a small volume of 2% SDS. Protein concentration was measured using the Detergent-compatible (DC) protein assay (Bio-rad). 5ug of each protein sample were loaded on SDS-PAGE at the appropriate concentration and transferred to nitrocellulose membrane (GE Healthcare). Western blotting standard protocols were then used to detect the indicated proteins, using the following antibodies. Primary antibodies: anti-LET-418 SZ4 (produced in our laboratory); anti-H3K27me3 07-449 (Merck-Millipore); anti-FLAG M2 (Sigma-Aldrich); anti-GAPDH (generous gift of A. Conzelmann); anti-total H3 #1791 (Abcam); anti-beta actin #A1978 (Sigma-Aldrich). Secondary antibodies: goat anti-rat-HRP, goat anti-Mouse-HRP, and anti-rabbit-HRP light-chain specific (Jacskon Immunoresearch, PA, USA). Western blots were revealed using enhanced chemo-luminescence (Thermo Scientific), and signals were detected using the Li-Cor Odyssee Fc system (Li-Cor). Quantification of band density was assessed with ImageJ Gel Analysis toolkit.

**Immunoprecipitations**

Immunoprecipitations (IPs) were performed using mixed embryonic pellets (100-200ul) of the indicated genotypes, as described previously [11]. Strep-tactin pull-downs were performed identically, using Strep-Tactin resin (IBA) instead of antibody-coupled beads. Western blots were generated and processed as described above. Antibodies used for this section, in addition to the list above, were the following: anti-LET-418 IP, #656 (produced in our laboratory) mixed with # 48960002 (NovusBiologicals).

**Additional Material References**

1. Brenner S: **The genetics of Caenorhabditis elegans**. *Genetics* 1974, **77**(1):71-94.

2. UniProt C: **UniProt: a hub for protein information**. *Nucleic acids research* 2015, **43**(Database issue):D204-212.

3. Edgar RC: **MUSCLE: multiple sequence alignment with high accuracy and high throughput**. *Nucleic acids research* 2004, **32**(5):1792-1797.

4. Letunic I, Bork P: **Interactive Tree Of Life v2: online annotation and display of phylogenetic trees made easy**. *Nucleic acids research* 2011, **39**(Web Server issue):W475-478.

5. Marchler-Bauer A, Derbyshire MK, Gonzales NR, Lu S, Chitsaz F, Geer LY, Geer RC, He J, Gwadz M, Hurwitz DI *et al*: **CDD: NCBI's conserved domain database**. *Nucleic acids research* 2015, **43**(Database issue):D222-226.

6. Kamath RS, Martinez-Campos M, Zipperlen P, Fraser AG, Ahringer J: **Effectiveness of specific RNA-mediated interference through ingested double-stranded RNA in Caenorhabditis elegans**. *Genome biology* 2001, **2**(1):RESEARCH0002.

7. Pfaffl MW, Tichopad A, Prgomet C, Neuvians TP: **Determination of stable housekeeping genes, differentially regulated target genes and sample integrity: BestKeeper--Excel-based tool using pair-wise correlations**. *Biotechnology letters* 2004, **26**(6):509-515.

8. Celniker SE, Dillon LA, Gerstein MB, Gunsalus KC, Henikoff S, Karpen GH, Kellis M, Lai EC, Lieb JD, MacAlpine DM *et al*: **Unlocking the secrets of the genome**. *Nature* 2009, **459**(7249):927-930.

9. Shen L, Shao N, Liu X, Nestler E: **ngs.plot: Quick mining and visualization of next-generation sequencing data by integrating genomic databases**. *BMC genomics* 2014, **15**:284.

10. Pourkarimi E, Greiss S, Gartner A: **Evidence that CED-9/Bcl2 and CED-4/Apaf-1 localization is not consistent with the current model for C. elegans apoptosis induction**. *Cell death and differentiation* 2012, **19**(3):406-415.

11. Kaser-Pebernard S, Muller F, Wicky C: **LET-418/Mi2 and SPR-5/LSD1 cooperatively prevent somatic reprogramming of C. elegans germline stem cells**. *Stem cell reports* 2014, **2**(4):547-559.
